# A Modified Yeast Two-Hybrid Platform Enables Dynamic Control of Expression Intensities to Unmask Properties of Protein–Protein Interactions

**DOI:** 10.1021/acssynbio.2c00192

**Published:** 2022-07-27

**Authors:** Erez Feuer, Gil Zimran, Michal Shpilman, Assaf Mosquna

**Affiliations:** The Robert H. Smith Institute of Plant Sciences and Genetics in Agriculture, The Hebrew University of Jerusalem, Rehovot 7610000, Israel

## Abstract

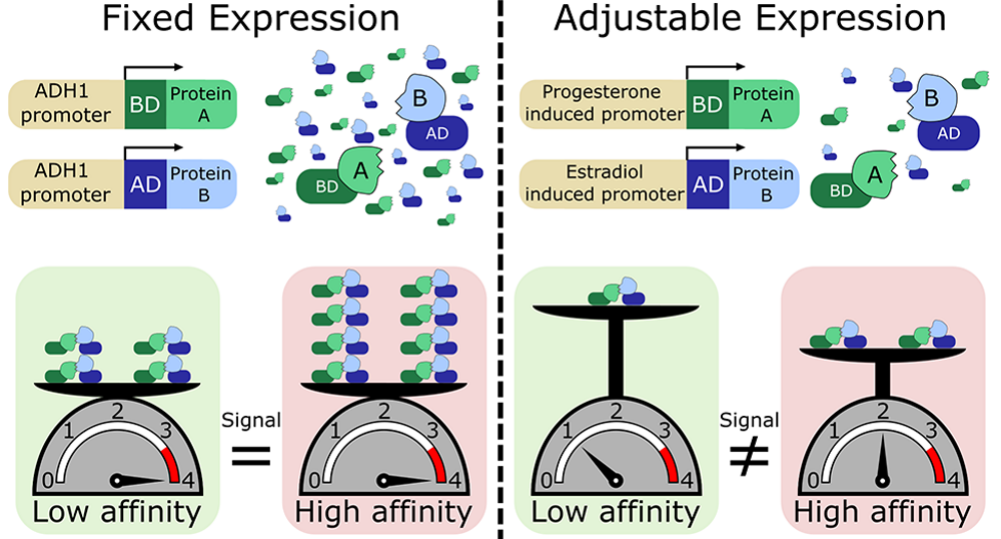

The yeast two-hybrid (Y2H) assay is widely used for protein–protein interaction characterization due to its simplicity and accessibility. However, it may mask changes in affinity caused by mutations or ligand activation due to signal saturation. To overcome this drawback, we modified the Y2H system to have tunable protein expression by introducing a fluorescent reporter and a pair of synthetic inducible transcription factors to regulate the expression of interacting components. We found that the application of inducers allowed us to adjust the concentrations of interacting proteins to avoid saturation and observe interactions otherwise masked in the canonical Y2H assay, such as the abscisic acid-mediated increase in affinity of monomeric abscisic acid receptors to the coreceptor. When applied in future studies, our modified system may provide a more accurate characterization of protein–protein interactions.

## Introduction

In biological systems, proteins commonly rely on interactions with other proteins to perform their function. These protein–protein interactions (PPIs) serve as the basis for fundamental biological processes such as post-translational modification and signal transduction. Although considerable attention is directed to discovering interaction networks and answering the question “who interacts with whom?”, numerous biological processes are driven by the nonbinary dynamics of PPIs. Many factors can affect PPIs, such as allosteric regulators, allelic variation, and the presence of mediating proteins, which may alter the affinity between a pair of proteins. Sometimes slight affinity shifts are enough to significantly influence biological processes.^1^ As current high-throughput assays produce greater volumes of data providing whole interactomes for different organisms, the need to better understand the dynamics of PPIs has become critical.

The yeast two-hybrid (Y2H) assay is a leading method for studying PPIs.^2,3^ In this assay, a pair of proteins fused to the GAL4 DNA binding domain (BD) and activating domain (AD) are expressed in yeast cells. Interaction between the two proteins reconstitutes a transcription factor and activates the transcription of a reporter gene. The fact that Y2H is based on a fast-growing microorganism host enables robust large-scale screens utilizing basic lab equipment. Y2H is commonly used for drug discovery and screening of PPI’s, in particular since Y2H libraries have become publicly available. In many aspects the Y2H is an excellent system; it has facilitated discoveries while being simple, robust, and compatible with proteins from different organisms. However, when applied to the characterization of a specific protein, there are two major drawbacks: limited information on kinetics and limited signal resolution.^3,4^ The latter becomes apparent when comparing protein family members, mutants, and allosteric changes. As in many detection systems, the utility of Y2H is determined by sensitivity and dynamic range.^5^ In molecular terms, sensitivity is the ability to detect interactions, and dynamic range is the ability to differentiate interaction strengths. Previous studies have shown Y2H is sensitive enough to detect interactions with *K*_d_ in the μM range, which enables it to cover a significant variety of biological PPIs.^4^ This high sensitivity is ideal for the detection of interactions, but when it is combined with a limited dynamic range, the assay’s resolution may be compromised by saturation. That is because a given affinity interaction may saturate the signal and thus appear identical with higher-affinity interactions, theoretically masking changes of a few folds in *K*_d_. In such cases, to differentiate between degrees of interaction affinities, saturation must be avoided. This can be achieved by adjusting the sensitivity or the dynamic range of the assay.

Many improvements have been introduced since the innovation of Y2H, to widen its application.^3^ Recently, several attempts were made to produce quantitative information via yeast interaction assays.^6−9^ Still, to the best of our knowledge, a Y2H variant that provides a comprehensive platform for saturation avoidance has not been widely adopted. Possibly the cumbersome nature of the solutions or availability of the needed equipment limits the application of such variants. Adjusting the sensitivity or dynamic range in these systems requires multilayer integration of several yeast strains with educated plasmid/reporter selection.^3,4,10^ This can be inconvenient, especially in situations where multiple comparisons are necessary. Thus, we aspired to create an “all-in-one” general system in which sensitivity could be easily fine-tuned; all the above manifested in one yeast strain, utilizing minimal expression vectors and basic lab equipment.

**Figure 1:**
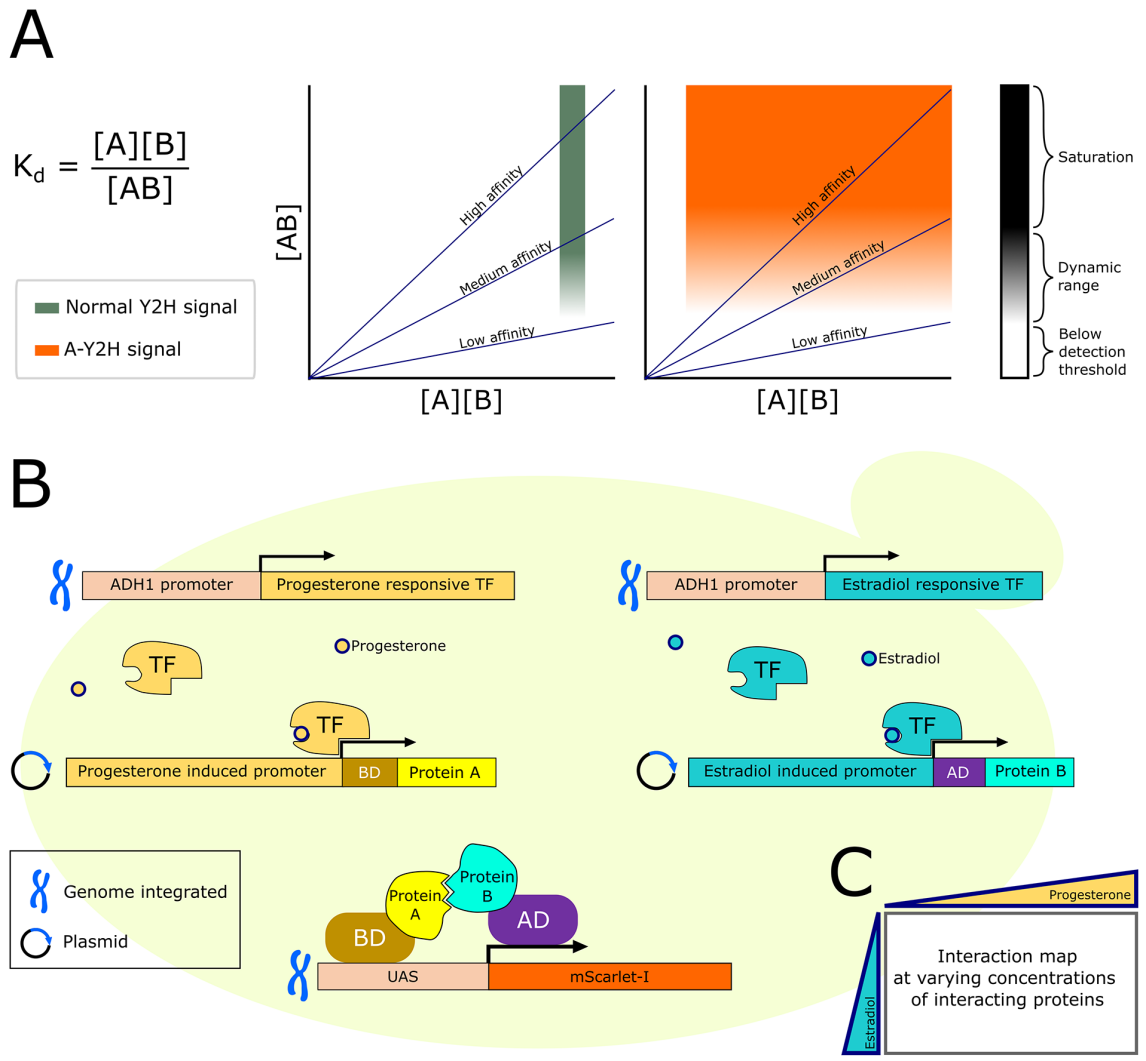
Adjustable yeast-two-hybrid (A-Y2H). (A) An illustrated model describing the signal dynamics of Y2H and A-Y2H at varying affinities and protein concentrations according to the *K*_d_ equation. (B) Schematic illustration of the A-Y2H system. Constitutive genomically expressed transcription factors (TF) activate Y2H cassette expression in response to progesterone or estradiol. Interaction between expressed proteins activates expression of mScarlet-I fluorescent reporter. (C) Scheme of an interaction matrix–output of the A-Y2H assay.

We hypothesize that expanding the concentration range of interacting proteins within the yeast cells will allow generation of unsaturated interaction signals, thus enabling greater differentiation between affinities (Figure 1A). According to the dissociation constant (*K*_d_) equation, derived from the law of mass action, in static cellular protein concentrations, equilibrium is reached and the concentration of PPI complexes becomes a function of the affinity (*K*_d_) and the concentrations of free apo-proteins.^11,12^ The expression of Y2H bait and prey proteins is traditionally driven by strong constitutive promoters such as ADH1 putatively resulting in fixed protein concentrations.^10^ For these reasons, we assume that in this assay the concentration of complexes determines the intensity of the output signal, which is relatively fixed and high.^4^ Consequently, above a certain concentration saturation begins to take place and the signal remains the same, although the concentration of PPI complexes rises. This leaves to chance the ability of Y2H to differentiate between two affinity states, hoping that at least one of the compared interactions has a concentration of PPI complexes that is below saturation level. Our hypothesis is based on the idea that adjusting the cellular concentrations of the interacting proteins can affect the concentration of PPI complexes according to the *K*_d_ equation and enable fine-tuning of the assay to avoid saturation (Figure 1A).

In this study, we developed a Y2H variant in which the expression of quarry protein is governed by chemically induced synthetic transcription factors. By titration of inducers, the cellular concentrations of the interacting proteins can be adjusted accordingly, thus facilitating a tunable range of output signals within the same strain of yeast. To optimize this system, we used a large protein family in which affinity can be modulated by a small molecule ligand. Lowering expression of the interacting proteins has enabled us to observe ligand-induced affinity changes previously masked by saturation of the classical Y2H signal. Using this system, we were able to show a ligand-mediated increase in the affinity of the tomato gibberellic acid (GA) receptor SiGID1a to SiPROCERA—previously unattainable information. We believe that the modifications we introduced will enable improved affinity resolving abilities while not compromising the simplicity and accessibility of the original assay, thus broadening its application.

## Results and Discussion

### Adjusting the Concentrations of Interacting Proteins in Yeast-Two-Hybrid Enables Sensitive Detection of Changes in Affinity

One of the main reasons Y2H is widely used in research is the fact that it is simple and accessible for most laboratories with basic “household” lab equipment and technical skills. We aspired to address some of the Y2H drawbacks by modifying Y2H without compromising its simplicity, since maintaining the system’s accessibility was a high priority for us. Therefore, we maintained a two-plasmid-single-yeast strain platform, and data acquisition using common lab equipment.

### Adjustable Yeast-Two-Hybrid System

To test our hypothesis, we decided that an inducible expression is a preferable strategy for modulating the expression of the Y2H interacting proteins because it enables fine-tuning. In yeast, there are a few endogenous inducible promoters, the most commonly used being GAL1, GAL7, GAL10, and CUP1.^13,14^ GAL1/7/10 originally drive galactose metabolism pathway proteins and are activated by the GAL4 transcription factor, and therefore are not compatible with Y2H strains that contain a deletion in the GAL4 locus.^2,13^ CUP1 is less suitable as well, due to high basal expression levels and the impact of Cu^2+^ ions on cellular structure and metabolism.^14,15^ This prompted us to use an exogenous induction system utilizing chimeric transcription factors (TF) adopted from two studies.^16,17^ These TFs are “synthetic nuclear receptors” consisting of a DNA binding domain, an allosteric inducer binding site, and an activating domain, enabling a range of transcriptional activation in response to inducer concentrations. We selected two different TFs that could be used to independently induce the expression of the two Y2H cassettes, one TF (LexA-ER-haB112) activated by the application of the estradiol and the other (ZPM) activated by progesterone. The TF expression cassettes were transformed into the genome of the Y2H strain Y190 under the regulation of the strong constitutive promoter ADH1. To incorporate this synthetic induction system, we replaced the plasmids’ promoters driving the AD and BD cassettes to promoters bearing the corresponding TF binding sites. Thus, expression of AD and BD fusion proteins is now driven by the estradiol or progesterone-induced-TF, respectively. After these modifications, we hypothesized that application of the inducers, estradiol and progesterone, would afford independent expression regulation of the AD and BD fusion proteins in a dose-dependent manner (Figure 1B,C). To test the activity of the synthetic induction system, AD and BD proteins were fused to mScarlet-I^18^ and expression levels were measured via fluorescence. Upon inducer application, both cassettes showed an increase in expression in response to increased estradiol or progesterone concentrations, respectively (Figure S1A,B). In addition, we evaluated expression by immunoblotting of three BD fused proteins tagged with FLAG (Figure S1C). All three proteins demonstrated an increase in expression correlated to increasing progesterone concentrations. We also noticed that under the same induction conditions the relative expression level of different proteins varied: PYR1, high; PYL4, intermediate; and PYL7, low. These results indicate that although expression induction works for various proteins, the expression level at identical inducer concentrations may drastically diverge for different proteins. While uneven expression across different proteins could impede their comparison, matching protein accumulation by differential induction could be a solution and quantification can be done by using a standard protein (Figure S1D). In addition to modifying the expression regulation of the interacting proteins, we added the fluorescent protein mScarlet-I as a reporter gene for easier acquisition of quantitative results.^18,19^ The new yeast strain together with the modified Y2H cassettes facilitates the culturing of a series of cells with gradually increasing concentrations of the interacting proteins (Figure 1B,C). Using this system requires minimal intervention and potentially enables quantitative data acquisition. From this point, we refer to it as adjustable yeast two-hybrid (A-Y2H).

### Demonstrating the Advantages of Adjusting Protein Concentrations Using a Family of Biochemically Diverse Receptors

We hypothesized that the modified Y2H capable of adjusting protein concentrations will outperform Y2H in cases where changes in affinity may be masked by signal saturation. This hypothesis was evaluated by characterizing the interaction between Pyrabactin Resistance 1/PYR1-like/Regulatory Component of ABA Receptor (PYR/PYL/RCAR) proteins and their coreceptors from the type 2C phosphatases (PP2Cs) family, which together serve as an abscisic acid (ABA) perception apparatus of *Arabidopsis thaliana*.^20,21^ One of the advantages of using ABA receptors is that the interaction of the same proteins can be tested while varying the affinity by ABA application, putatively avoiding influences generated by protein-related differences (e.g., protein stability, expression levels, or folding). Previous *in vitro* studies show that binding of ABA to the receptors induces an active conformation, in which their affinity to the coreceptor increases.^20,22−27^ The receptor family comprises two distinct classes of receptors that vary in affinity to the coreceptors in the absence of ABA activation (basal activity): a class of dimeric receptors that lack basal activity, and a class of monomeric receptors that have a higher basal activity. Y2H, in its current form, is incapable of detecting the affinity changes of the monomeric receptor. For those receptors, the Y2H interaction signal remains the same with or without ABA.^20,21,28^ These results do not coincide with *in vitro* assays where the presence of ABA affects receptor–coreceptor interaction.^29^ We found this contradiction ideal to assess the aforementioned hypothesis.

**Figure 2:**
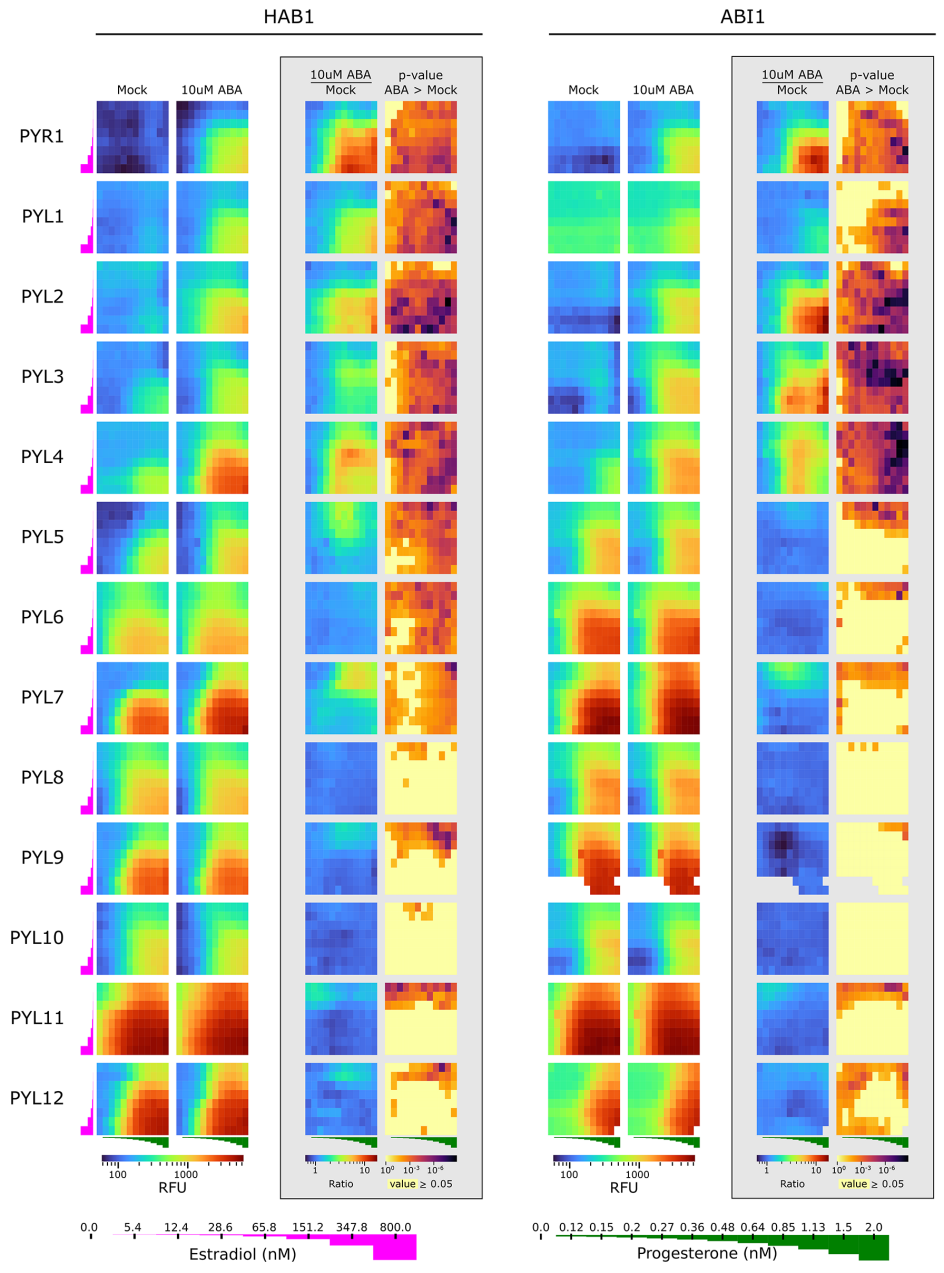
A-Y2H results for the interaction of 13 ABA receptors vs HAB1 and ABI1. Analysis of mScarlet-I fluorescent signal from interactions at a matrix of increasing concentrations of progesterone (0–2 nM) and estradiol (0–800 nM) in the presence and absence of ABA. The receptors and HAB1/ABI1 were expressed from pBD-pZ-FLAG and pACT-Lex, accordingly, in a Y190 strain containing genomic integrations of UAS::mScarelt-I and inducting TFs. Heatmaps display the signal of mock (0.1% DMSO) (left), with ABA (center-left), the ratio between the signals of the two states (center-right), and the statistical significance of the ABA-mediated increase in signal as the *p*-value of *t* test assuming unequal variances (right), for each combination of progesterone and estradiol concentrations. Missing values such as seen in PYL9/PYL12 vs ABI1 are due to OD filtering as described in the Methods section. *n* = 3–4. The same data for PYR1-HAB1, PYL5-HAB1, and PYL10-HAB1 appear also in Figure 3 or Figure S3.

**Figure 3:**
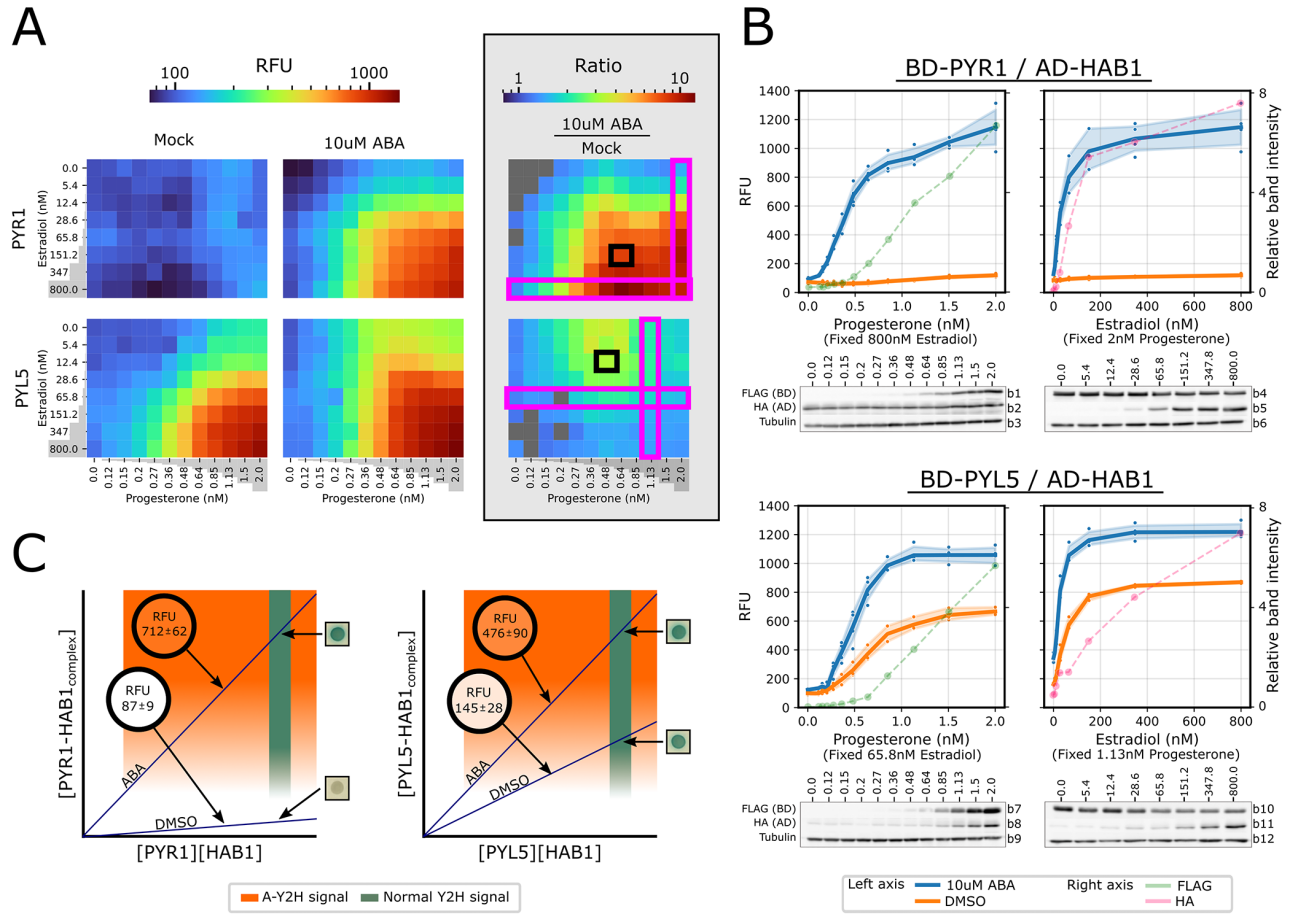
Resolving signal saturation by adjusting Y2H protein levels. Y2H signal saturation masked ABA-mediated change in affinity of PYL5 to HAB1; lowering the cellular concentrations of the interaction counterparts enables detection of these changes. (A,B) mScarlet-I fluorescent signal from interactions of PYR1 or PYL5 with HAB1 at a matrix of increasing concentrations of progesterone (0–2 nM) and estradiol (0–800 nM) in the presence and absence of ABA. PYR1/PYL5 and HAB1 were expressed from pBD-pZ-FLAG and pACT-Lex, accordingly, in a Y190 strain containing genomic integrations of UAS::mScarelt-I and inducting TFs. *n* = 3–4. The same fluorescent data appears in Figure 2 (A) Heatmaps displaying the signal of mock (0.1% DMSO) (left), with ABA (center), and the ratio between the signals of the two states (right) for each combination of progesterone and estradiol concentrations. Results present means of four technical repetitions for each different combination of proteins, inducers, and ligand. Areas marked in pink and black correspond to progesterone and estradiol concentrations in (B) and (C), respectively. Gray-colored matrix cells indicate instances in which ABA presence did not produce a significantly (*p*-value > 0.05) higher signal according to *t* test assuming unequal variances. (B) Curves at selected progesterone and estradiol concentrations along matching Western blots performed on protein extractions of the same yeast cell used for RFU measurements. The curve displaying relative band intensity is of the protein that is regulated by the chemical on the *X*-axis (FLAG, progesterone; HA, estradiol). ABA and mock yeast wells were combined for protein extraction. Dots represent all measurements taken. Colored curve bands represent 0.95 confidence. Uncropped Western blot results can be observed in the Supporting Information according to the numbering to the right of each blot (Figure S4). (C) Hypothetical model demonstrating how adjusting the concentrations of interacting proteins enables differentiation of interaction affinities. Squares to the right of each plot are actual X-gal staining Y2H results for the specific interactions. Results in the black circles are of A-Y2H produced RFU signals at selected estradiol and progesterone concentrations as noted in (A).

In this study, we used A-Y2H to characterize combinations of 13 receptors (PYR1 and PYL1–12) vs two PP2C (HAB1 and ABI1) in two activity modes: lower affinity, apo state; and higher affinity, ligand-bound (Figure 2 and Figure 3). The assay for every activity mode was carried out in a series of graded inducers concentrations (estradiol and progesterone). This enabled us to observe interactions of all the above combinations in a matrix of expression levels. The matrix can be later used to focus on fine-tuned estradiol-progesterone concentrations that reveal additional information. To better observe the ligand effect, the signal of ABA treated samples was divided by that of the mock for every combination of inducer concentrations within the matrix, thus creating a new matrix where the effect of ABA presence on the interaction can be analyzed using statistical tools (Figure 2 and Figure 3A). Characterizing the dimeric receptors (PYR1, PYL1/2) shows that in their active form (in the presence of ABA), the receptors displayed an increase in interaction signal in response to increasing inducer concentrations. The inactive form produced a low background signal that did not increase upon inducer application. In these results, the effect of ABA on the interaction is apparent as in the unmodified Y2H, thus confirming that the modulations made in protein expression do not impair the abilities of Y2H to detect interactions within the micromolar range^30^ (Figure S2). It was apparent that for seven of the nine monomeric receptors, A-Y2H was able to detect an increase in interaction signal in the presence of ABA, contrary to Y2H results^20^ (Figure 2). The ratio between the signals of the two states revealed a pattern in which the increase, sometimes of a few folds (for example PYL7 with a maximal ratio of 4.9), was most significant at lower inducer concentrations, while at high inducer concentrations it was not as significant (Figure 2). This reinforces our hypothesis that adjusting protein concentrations can overcome signal saturation and unmask important information. At low inducer concentrations, fewer proteins are in an interaction complex resulting in a lower, unsaturated output signal, which in turn enables distinguishing between apo and ligand-bound monomeric receptors. We could not differentiate between the two aforementioned states for all monomeric receptors, for example PYL10, for which the presence of ABA did not display a significant change in interaction signal (Figure S3). *In vitro* studies showed PYL10, in comparison to other PYLs, has high basal activity that is sufficient to elicit signaling in plants independently of ABA.^30−32^ We believe that the change in affinity by ABA, although actual, is not as significant for PYL10 and therefore is challenging to detect by the resolution of our system. Of the 13 ABA receptors tested, we chose to showcase a dimeric receptor (PYR1) and a monomeric receptor (PYL5) because they represent opposed basal activities: low and high, respectively^33^ (Figure 3). The affinity increase of PYR1 to PP2C as a result of the presence of ABA spans across both sides of the detection threshold of Y2H and is, therefore, detectable in that system (Figure 3C). On the other hand, PYL5 has high basal affinity that produces a saturated signal in Y2H, so the increase in affinity by ABA is unnoticeable, as it produces the same saturated signal (Figure 3C). Being able to adjust the protein concentrations enabled observing situations in which the output signal is unsaturated and within the dynamic range of the assay. This allowed us to better differentiate between interaction affinities and perceive the effect of ABA on PYL5 as illustrated in the hypothetical model (Figure 3C).

### Demonstrating Comparability with Other Proteins: Gibberellic Acid Receptors

**Figure 4:**
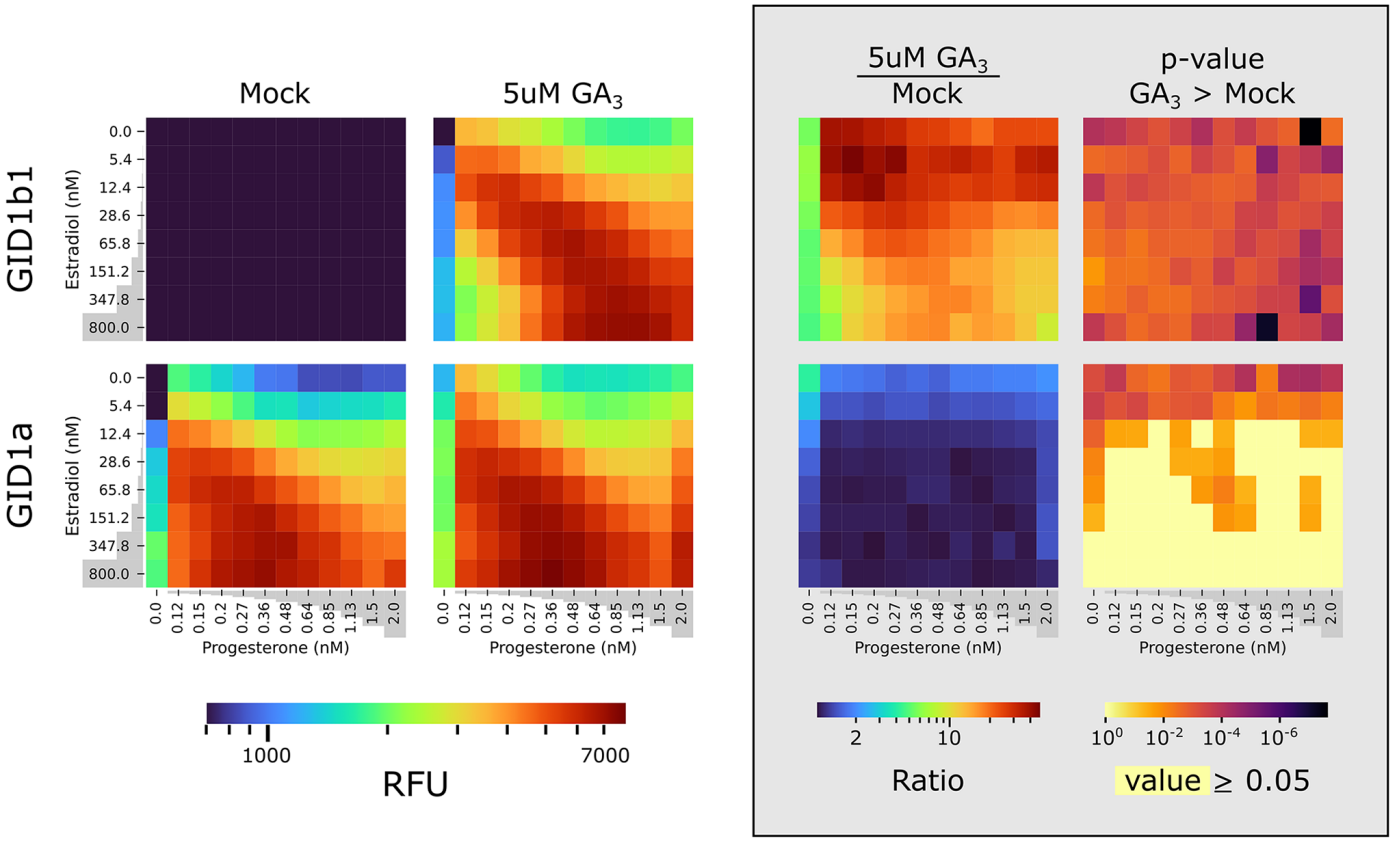
A-Y2H reveals the gibberellic acid receptor SiGID1a has high basal activity but is GA induced. Analysis of mScarlet-I fluorescent signal from interactions of SiGID1b1 or SiGID1a with SiPROCERA at a matrix of increasing concentrations of progesterone (0–2 nM) and estradiol (0–800 nM) in the presence and absence of GA_3_. GID1b1/GID1a and PROCERA were expressed from pBD-pZ-FLAG and pACT-Lex, accordingly, in a Y190 strain containing genomic integrations of UAS::mScarelt-I and inducting TFs. Heatmaps display the signal of mock (0.1% DMSO) (left), with GA_3_ (center-left), the ratio between the signals of the two states (center-right), and the statistical significance of the GA_3_-mediated increase in signal is expressed as the *p*-value of *t* test assuming unequal variances (right), for each combination of progesterone and estradiol concentrations. *n* = 3.

As we aspired to maintain the essence of Y2H, it was important for us to verify the compatibility of A-Y2H with different proteins. Therefore, we used A-Y2H to characterize the interaction of two gibberellin receptors from tomatoes (Figure 4). It was previously shown in Y2H that the gibberellic acid (GA) receptors SiGID1b1 and SiGID1a interact with SiPROCERA in a GA-dependent and a GA-independent manner, respectively.^34^ Using A-Y2H we were able to reproduce the Y2H results and gain additional information regarding the effect of gibberellin on SiGID1a. When comparing the Y2H results of Illouz-Eliaz *et al.* (2019)^34^ with our A-Y2H results, we found that in both systems, SiGID1b1 displayed full dependency on GA presence for interaction with SiPROCERA. Analysis of SiGID1a using A-Y2H showed that it is also affected by the presence of GA (Figure 4). Furthermore, the GA-mediated increase in affinity of SiGID1a was most significant at lower induction levels of protein concentrations, similar in trend to the ABA-mediated increase in affinity of the monomeric ABA receptors. This suggests that SiGID1a, like the monomeric PYL receptors, has basal ligand-independent activity that is further increased by ligand binding. To the best of our knowledge, the effect of GA on SiGID1a was never observed *in vitro*; hence, there is no evidence to corroborate our finding. However, this demonstrates how A-Y2H can excel in providing additional, previously unattainable information, which is important for understanding the mechanics of the signaling systems.

### Can Yeast Assays Replace *in Vitro* Binding Assays?

In classic biochemical *in vitro* assays, affinity is commonly deduced from a binding curve, in which one of the interacting proteins is titrated while the second is maintained at a constant concentration.^12^ At the start, the titrated protein is a limiting factor, and the signal increases with the progress of titration. When the titrated protein is no longer a limiting factor, the signal reaches a plateau, as no more units of the constant protein are available for interaction.^12^ In A-Y2H a similar curve can be achieved as the expression of one protein increases by inducer titration, while the expression of the other protein is kept at a constant induction level (represented by a row or column in the matrix of inducer concentrations) (Figure 3B and Figure S2B). To ensure that inducer-mediated titration facilitates protein accumulation beyond the saturation point, we performed immunoblotting for HAB1, PYR1, PYL5, and PYL10 on selected rows/columns (Figure 3B and Figure S2B). Results showed that expression induction had not reached a plateau at the point at which the interaction signal plateaus—rather, it continued to rise as the inducer concentration increased. In most cases, the rise in protein accumulation due to induction was linear with a magnitude of a few folds across the saturated signal, thus confirming that the induction system is not a limiting factor, and saturation is not caused by stationary protein expression.

Although the circumstance and essence of the obtained curve resemble that of an *in vitro* binding curve underlying biochemical information, there is a great difference between *in vitro* and assays in living cells that needs consideration. In our cellular-based system, many more factors are involved between the point of expression of a given protein and up to the generation of an output signal, factors that complicate absolute quantification of interaction parameters.^4^ To name just a few: protein expression interlinkage, transportation efficiency of interacting proteins to the nucleus, susceptibility to proteolysis, protein aggregation, and interaction with endogenous protein of the host organism.^35^ In our immunoblot results, we noticed in some cases interlinkage in the accumulation of the two proteins. For example, the expression of progesterone-induced PYL10 decreased as estradiol-induction of HAB1 increased (Figure S2B). Although our system can not fully replace *in vitro* binding assays, it is still benefiting from a higher resolution of titration binding assays and from the scalability of genetically coded systems, which are cost-effective both in resources and technical skills.

## Conclusion

Saturation is a weak link of every detection system when trying to acquire quantitative results. Information derived from a saturated signal does not correctly represent the true situation, and therefore it is susceptible to error.^5^ An everyday example would be an overexposed landscape image. During overexposure, both the sky and the sun emit enough light to reach the limit of light the camera sensor can sense, which causes a saturated signal. In this image, they will appear the same and it would be impossible to gain information such as the location of the sun in the sky. We hypothesized that the signal obtained in Y2H for monomeric ABA receptors is saturated and that avoiding saturation will enable detection of the ABA-mediated increase in affinity for the coreceptor. Our strategy to avoid saturation was to adjust the cellular concentrations of the interacting proteins to lower levels by induction.

In the modified Y2H system, changing protein expression levels by induction altered the output signal generated by interaction. In most cases, an increase in the concentration of one of the inducers increased the interaction signal. Thus, we believe the signal had not reached saturation, indicating that protein-expression-tuning within A-Y2H effectively prevents saturation. Utilizing this system enabled us to observe information previously masked in Y2H by saturation such as the ABA and GA-mediated increase in affinity of their corresponding receptors to the coreceptor. The system we created maintains the essence of Y2H: it is simple, accessible, and has the same two-plasmid-single-yeast strain architecture, yet it provides more information. As it is compatible with other PPIs, we believe it can become a “household” assay for many applications.

## Methods

### Plasmid Construction

All vector assembly was done using the homology-based Gibson assembly method.^36^ Fragments were amplified by PCR with homology sequences (Figure S5, Figure S6, Table S2, and Table S3) with primers ordered from IDT (IA, USA). All vectors were linearized by restriction enzymes by New England Biolabs (MA, USA) (Table S1). We transformed constructed plasmids into the *E. coli* strain DH5α by heat shock. All ABA and GA pathway proteins were amplified from cDNA of *A. thaliana* (Col-0) and *S. lycopersicum* (M82) respectively. PYR1 (AT4G17870), PYL1 (AT5G446790), PYL2 (AT2G26040), PYL3 (AT1G73000), PYL4 (AT2G38310), PYL5 (AT5G05440), PYL6 (AT2G40330), PYL7 (AT4G01026), PYL8 (AT5G53160), PYL9 (AT1G01360), PYL10 (AT4G27920), PYL11 (AT5G45860), PYL12 (AT5G45870), GID1a (Solyc01g098390), GID1b1 (Solyc09g074270), and mScarlet-I were cloned into pBD-GAL4 (Clontech, CA, USA) or pBD-pZ-FLAG (Table S1) linearized with *Sal*I and *Eco*RI. HAB1 (AT1G72770), ABI1 (AT4G26080), PROCERA (Solyc11g011260), and mScarlet-I were cloned into pACT (Clontech, CA, USA), or pACT-Lex (Table S1) linearized with *Eco*RI NcoI.

### Yeast Strain Construction and Yeast Plasmid Transformations

All yeast strains developed in this work are based on the *Saccharomyces cerevisiae* strain Y190.^37^ Genomic integrations were done into two sites: HO locus^38^ and site 20.^39^ The former was used for integrations of the fluorescent reporter mScarlet-I and the latter was used for the integration of progesterone-induced and estradiol-induced TFs. The integrations were done by homologous recombination in which linear DNA fragments containing overlaps to the integration sites at the ends and antibiotic resistance were transformed as described in *Yeast Protocols*.^37^ Linear fragments used for integration were assembled in plasmids and were linearized by restriction enzyme digestion or PCR amplification (Figure S5, Figure S6, Table S1, and Table S2). Plasmids containing Y2H or T-Y2H cassettes were transformed into yeast as described in *Yeast Protocols*.^37^

### Media and Chemicals

All chemicals originate from Sigma-Aldrich (MO, USA) if not stated otherwise. For auxotroph-based selection, yeast was grown in a synthetic dextrose medium, lacking leucine or tryptophan as described.^37^ For antibiotic-based selection, yeast was grown on yeast extract–peptone–dextrose (YPD) medium, containing G418 or nourseothricin as described.^37^ DH5α was grown in standard lysogeny broth (LB) with ampicillin or chloramphenicol.

### X-gal Staining of Yeast

Y190 strains expressing Y2H cassettes were plated on synthetic dextrose medium lacking leucine and tryptophan and containing 10 μM ABA or 0.1% DMSO as a mock control. The plates were incubated for 2 days at 30 °C. The interaction was then visualized by X-gal staining, which indicates the enzymatic activity of the reporter β-galactosidase as described previously.^20^

### Relative Fluorescent Measurements of Yeast

Yeast strains expressing fluorescent reporters were prepared for fluorescent measurement in the following manner: 24 h before measuring fluorescence we prepared 150 μL of OD_600_ = 0.05 of yeast in synthetic dextrose medium with the appropriate chemical treatment (estradiol*/progesterone*/ABA/DMSO/GA3; *see paragraph below) for each well in a 96 well cell culture plate (Corning, NY, USD). The plate was then incubated at 30 °C while shaking at 1050 rpm (Titramax 100 by Heidolph Instruments, Germany) to avoid pelleting of the yeast. The plate was later used for fluorescent and OD readings using the Synergy H1 monochromatic fluorescence plate reader (BioTek Instruments, VT, USA). mScarlet-I was read from the bottom using 570/600 nm excitation/emission filters at a gain of 0. Fluorescent reads represent a mean of 10 measurements for each well. OD reads represent a mean of 8 measurements at 600 nm for each well. To obtain relative fluorescent units (RFU), fluorescent reads were normalized by dividing by the OD. Wells in which the OD did not reach 0.4 or exceeded 1 were filtered out, as we found that dividing by these ODs creates bias. Repetition numbers (*n*) mentioned in legends indicate the number of independent cell cultures of the same clone and chemical treatment (estradiol/progesterone/ABA/GA/mock) used for generating the average RFU. All data from the plate reader was analyzed using python code.

The need for high dilutions from the stock down to the nM range combined with a narrow induction range (small concentration changes result in high induction changes) creates large errors, thus introducing difficulties. We found it challenging to reproduce estradiol and progesterone concentrations for reoccurring induction. To overcome this challenge and perform the same induction levels across all our experiments we made a stock 96 well plate containing all progesterone and estradiol combinations at 30× concentrations. This plate was kept at −20 °C and used for all the experiments in this work containing estradiol and progesterone except for the GA receptors and experiment presented in Figures 4 and S1C; for those, different batches were made. The estradiol and progesterone concentrations used in this work were calibrated to cover the range between the signal at zero induction to signal at saturation. We anticipate that reproduction of the work presented in this paper will require recalibration of the concentrations of inducers due to inevitable error. We advise new users to calibrate for appropriate induction concentrations for their proteins by the initial application of a high range of inducer concentrations and later narrow down that range to obtain the optimal coverage of signal from minimum to saturation. As a side note, we noticed some leakiness in the estradiol-induced expression, as we got a low interaction signal in the absence of estradiol; this should be considered when designing an experiment based on this system.

### Immunoblotting

Following RFU measurements, total proteins were extracted from yeast in the 96 well plates for immunoblotting. The extraction method was adapted from Kushnirov.^40^ This method was proven to yield reproducible results with relative ease, which was convenient for the number of samples we handled. For the extraction, we combined the content of eight replicating wells (∼1200 μL) into a 1.7 mL tube and removed the supernatant after centrifuging for 2 min at 6000 rpm (centrifuge model). The pellet was then resuspended with 700 μL of cold water and subsequently centrifuged in the same manner. We then removed the supernatant, suspended the pellet with 300 μL of 0.1 M NaOH, vortexed the tube for five seconds, and incubated for 10 min at room temperature. The tube was then centrifuged at maximal speed for 1 min and the supernatant was removed. Next, we resuspended the pellet with 220 μL of SDS-sample-buffer (62.5 mM Tris-HCl pH 6.8, 2.5% SDS, 5% β-mercaptoethanol, 10% glycerol, and traces of Bromophenol Blue) and incubated it at 95 °C for 5 min. Following that the tubes were centrifuged for 1 min at maximal speed, and the supernatant was collected and stored at −20 °C for later use in immunoblotting. For quantification of FLAG-tagged samples as seen in Figure S2D, we used purified His10-FLAG-BRD4 (SP-600-100, R&D Systems, MN, USA) in SDS-sample-buffer as a standard. For Western blot analysis, the extracted protein and standard samples were incubated at 95 °C for 10 min and separated using SDS/PAGE separation followed by transfer to a nitrocellulose membrane. For FLAG, HA and tubulin detection, membranes were incubated overnight with the primary antibodies sc-166384 (Santa Cruz Biotechnology, CA, USA) at 1:1000, ab9110 (Abcam, MA, USA) at 1:4000, and ab184970 (Abcam, MA, USA) at 1:10 000, respectively. We used the secondary antibodies 111-035-003 (Jackson ImmunoResearch Inc., PA, USA), 115-035-003 (Jackson ImmunoResearch Inc., PA, USA), and the detection reagent NEL103001EA, Western LightningR Plus ECL (PerkinElmer, MA, USA). Images were acquired using the ImageQuant LAS 4000 mini (GE Healthcare, IL, USA) and analyzed with Fiji (ImageJ) (Figure S4).

## Supporting Information Available

Figure S1: Validation of the chemical induction system; Figure S2: A-Y2H results in comparison to known *K*_d_s; Figure S3: Showcasing PYL10 in A-Y2H; Figure S4: Immunoblot membranes used in this work, uncropped; Figure S5: Cloning procedure; Figure S6: Basic vector components for A-Y2H; Tables S1–S3: Cloning sequence information (PDF)
