# Supplementary material for: A Modified Yeast Two-Hybrid Platform Enables Dynamic Control of Expression Intensities to Unmask Properties of Protein–Protein Interactions
Source: ACS Synth Biol. 2022 Jul 27;11(8):2589–98. doi: 10.1021/acssynbio.2c00192 (PMC9442787; doi:10.1021/acssynbio.2c00192)
Supplement: Supplementary file 1 — sb2c00192_si_001.pdf [file sb2c00192_si_001.pdf]

Supplementary Information

Modified yeast two-hybrid platform enables dynamic control of expression intensities to unmask properties of protein-protein interaction

Erez Feuer, Gil Zimran, Michal Shpilman, Assaf Mosquna*

The Robert H. Smith Institute of Plant Sciences and Genetics in Agriculture, The Hebrew University of Jerusalem, Rehovot 7610000, Israel

*Corresponding author

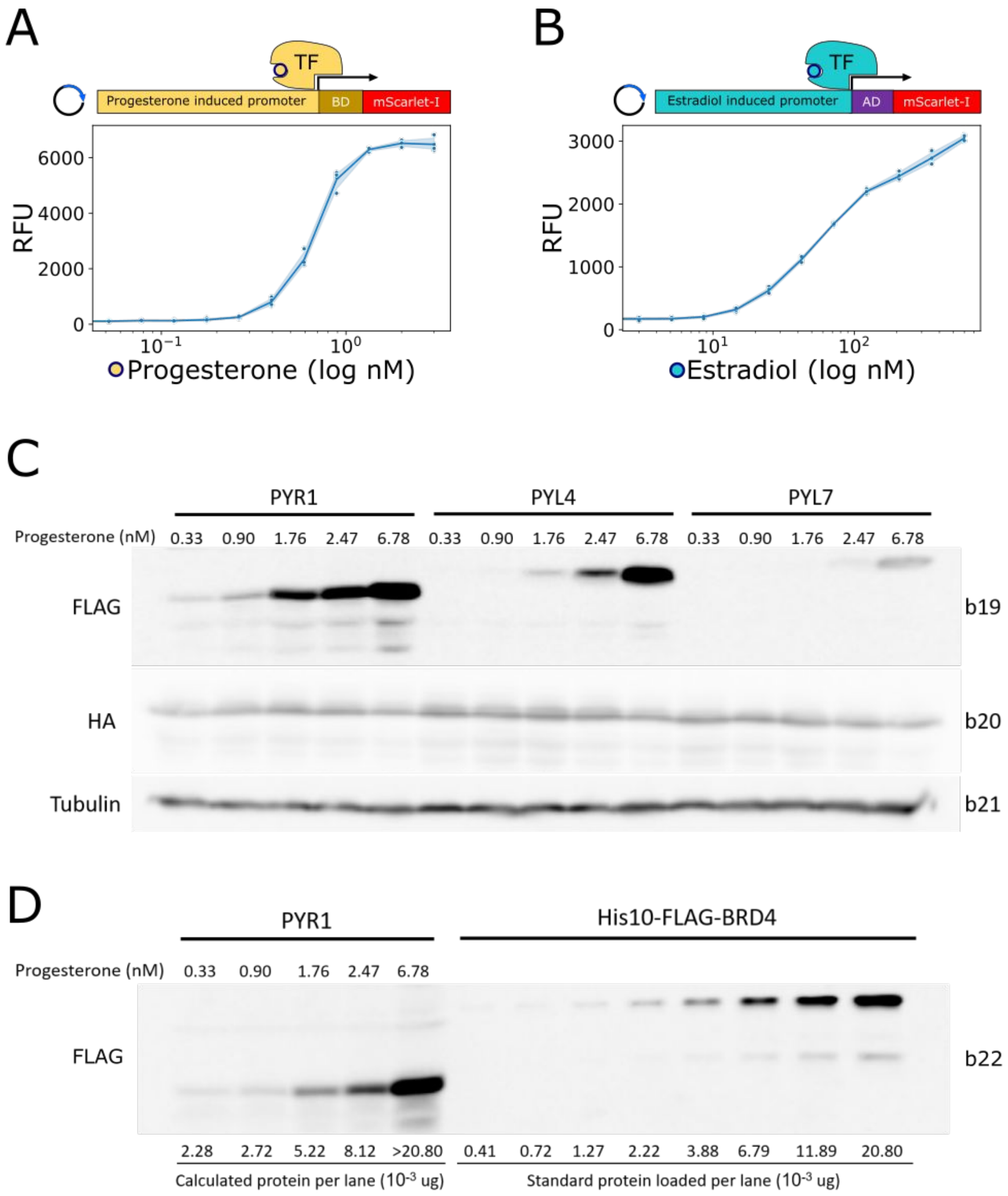

Figure S1. Protein accumulation gradually increases with inducer application. **(A,B)** Induction of Y2H cassettes measured by mScarlet-I fluorescence fused to Y2H proteins. Dots represent all measurements taken. Colored curve bands represent 0.95 confidence. n=4. **(A)** Induction of BD cassette with progesterone (at 0, 0.052, 0.078, 0.117, 0.176, 0.263, 0.395, 0.889, 1.333, 2 and 3 nM). **(B)** Induction of AD cassette with estradiol (at 0, 3, 5.1, 8.6, 14.6, 24.9, 42.3, 71.8, 122.1, 207.6, 352.9 and 600 nM). **(C)** Variation in protein accumulation for identical induction levels across different proteins. Western blot results for protein extraction of yeast expressing AD-HA-HAB1 and BD-FLAG-PYR1/PYL4/PYL7 after growth in 800nM estradiol and increasing levels of progesterone. **(D)** Quantification of BD-FLAG-PYR1 in yeast via western blot using purified His10-FLAG-BRD4 (SP-600-100, R&D Systems, MN, USA) as a standard. BD-FLAG-PYR1 samples used in this blot are from the same protein extraction presented in (c). Quantification normalized per 1 million cells for 0.33, 0.90, 1.76, 2.47 and 6.78 (nM) progesterone is 1.11, 1.37, 2.86, 4.51 and >10.12 (10^{-3}ug) respectively. Uncropped western blot results can be observed in the supplementary information according to the numbering to the right of each blot (Figure S4).

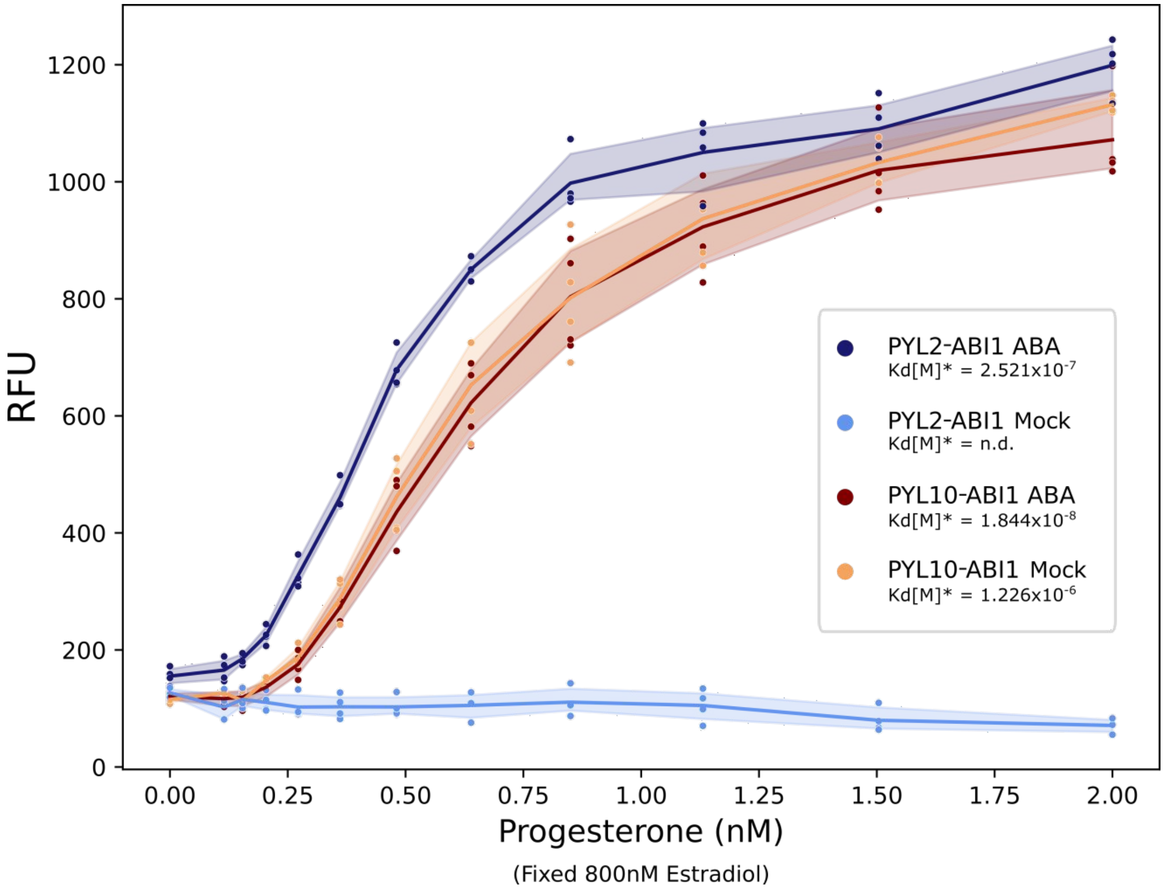

Figure S2. Adjustable-Y2H system can detect interactions within the micromolar range. A-Y2H results for the interactions of BD-PYL2/10 with AD-ABI1 in the presence and absence of ABA. *Kd values by Hao et al. 2011 (surface plasmon resonance). Dots represent all measurements taken. Colored curve bands represent 0.95 confidence.

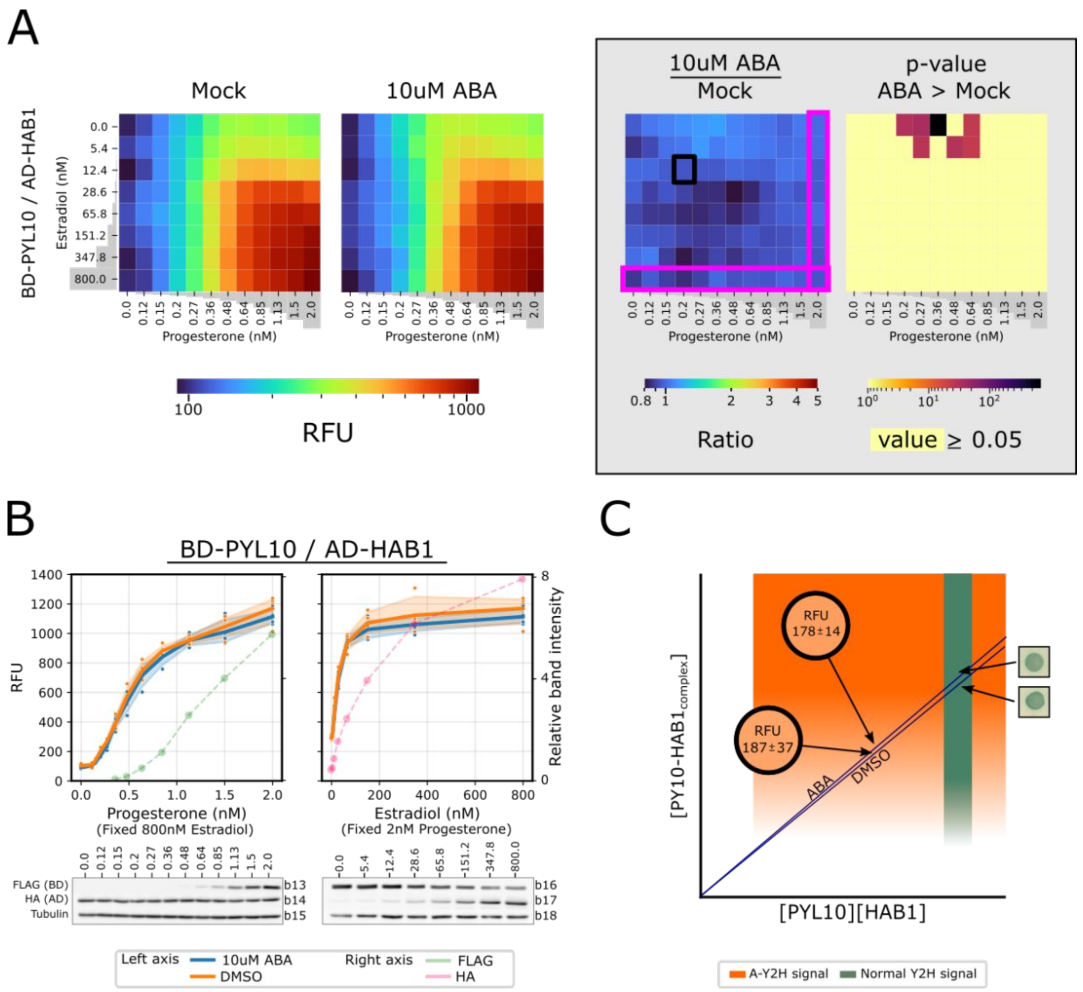

Figure S3. Adjustable-Y2H system could not resolve the ABA-mediated change in the affinity of PYL10 to HAB1. **(A,B)** mScarlet-I fluorescent signal from interactions of PYL10 with HAB1 at a matrix of increasing concentrations of progesterone (0-2 nM) and estradiol (0-800 nM) in the presence and absence of ABA. Pyl10 and HAB1 were expressed from pBD-pZ-FLAG and pACT-Lex, accordingly, in a Y190 strain containing genomic integrations of UAS::mScarelt-I and inducting TFs. n=4. The same fluorescent data appears in Fig. 2 **(A)** Heatmaps displaying the signal of mock (0.1% DMSO) (left), with ABA (center-left) the ratio between the signals of the two states (center-right) and the statistical significance of the ABA-mediated increase in signal as the p-value of t-test assuming unequal variances (right), for each combination of progesterone and estradiol concentrations. Results present means of four technical repetitions for each different combination of proteins, chemicals and ligand. Areas marked in pink and black correspond to progesterone and estradiol concentrations in (B) and (C), respectively. **(B)** Curves at selected progesterone and estradiol concentrations along matching western blots performed on protein extractions of the same yeast cell used for RFU measurements. The curve displaying relative band intensity is of the protein which is regulated by the chemical on the X-axis (FLAG – progesterone / HA – estradiol). ABA and DMSO yeast wells were combined for protein extraction. Dots represent all measurements taken. Colored curve bands represent 0.95 confidence. Uncropped western blot results can be observed in the supplementary information according to the numbering to the right of each blot (Fig. S3). **(C)** Hypothetical model demonstrating how adjusting the concentrations of interacting proteins enables differentiation of interaction affinities. Squares to the right of the plot are actual x-gal staining Y2H results for the specific interactions. Results in the black circles are of A-Y2H produced RFU signal at selected estradiol and progesterone concentrations as noted in (A).

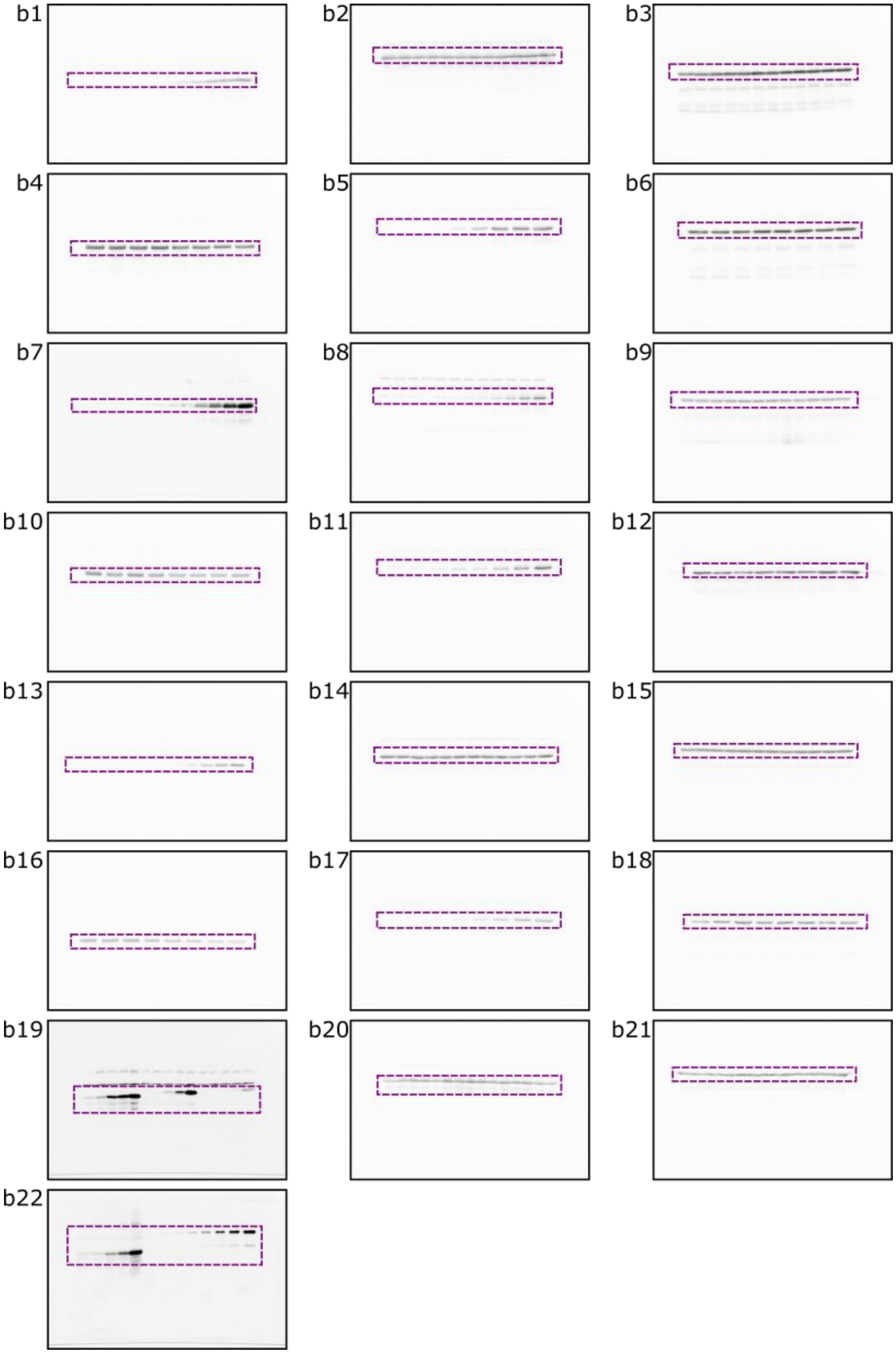

Figure S4. All western blot used in this work. Purple squares indicate the cropped area used in other figures according to the numbers on the top right corner of each membrane.

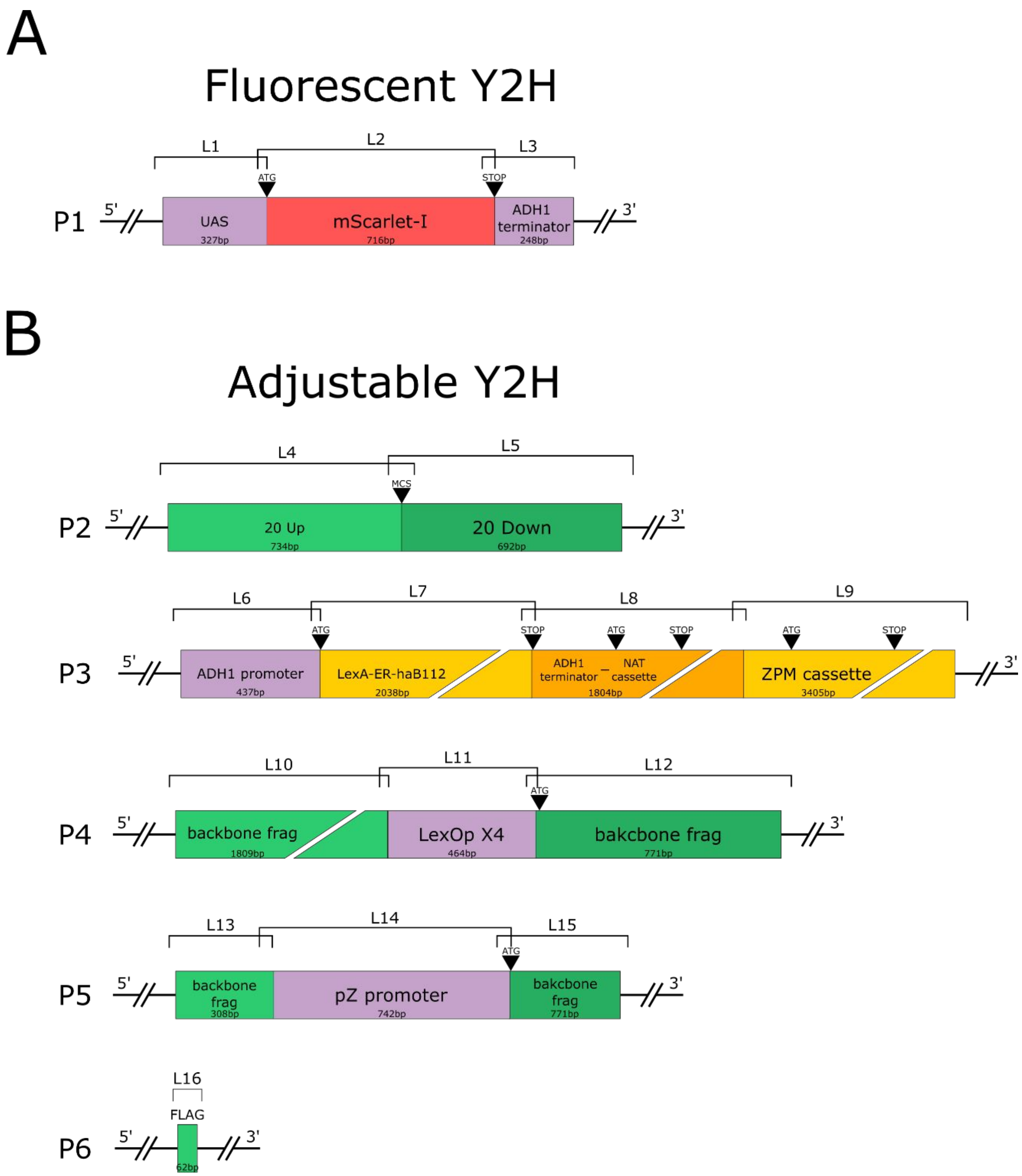

Figure S5. Vector assembly that was done in this work. P1-P7 represent plasmids and correspond with the information in Table S1. L1-L15 represent linear fragments and correspond with the information in Table S2 and Table S3. ATG represents the beginning of an open reading frame; STOP represents the ending of an open reading frame; MSC represents multi cloning sites. **(A)** Vectors use to add a fluorescent reporter to Y2H. **(B)** Vectors used in the construction of the Adjustable yeast two-hybrid system.

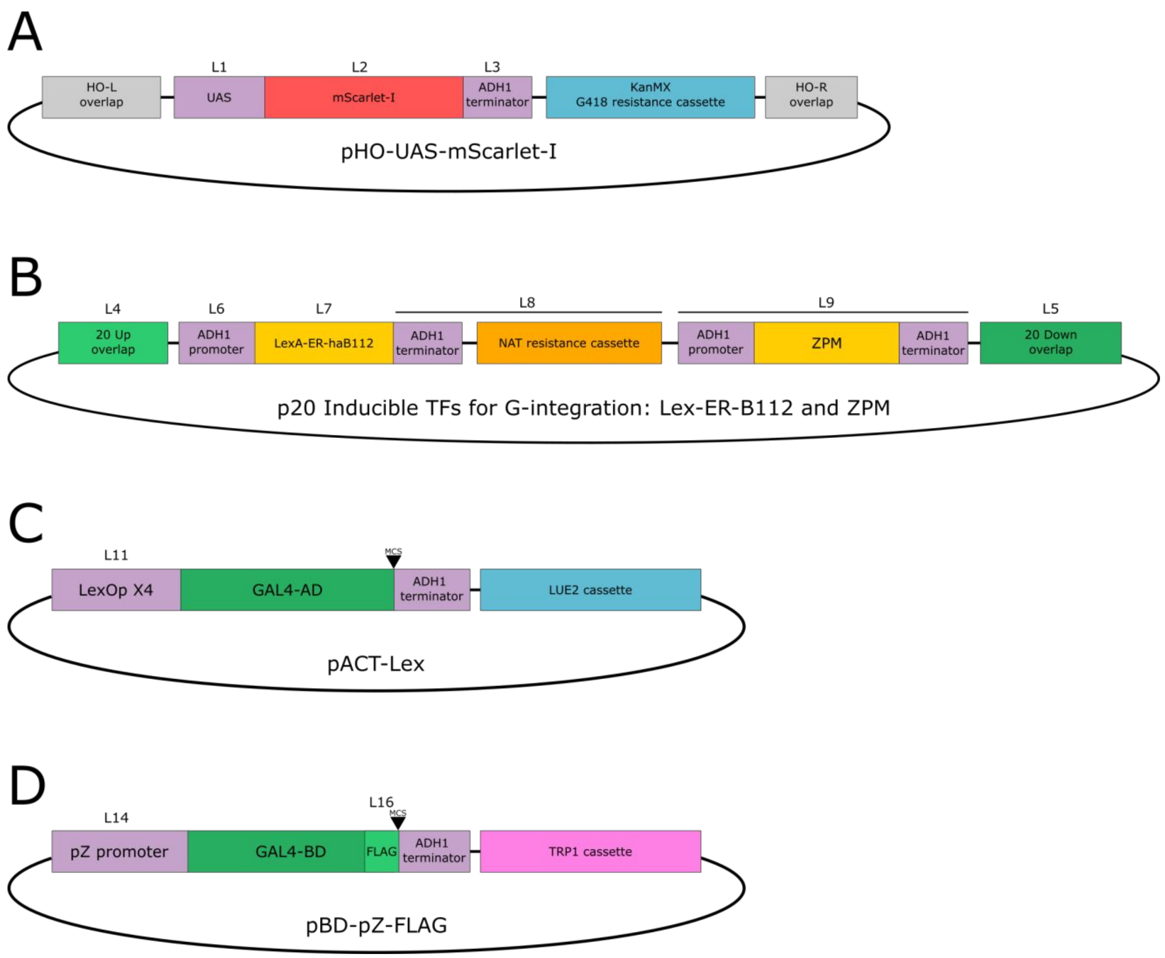

Figure S6. Adjustable-Y2H plasmids. (A) Plasmid for genomic integration of mScarlet-I as a reporter for yeast two-hybrid. **(B)** Plasmid for genomic integration of estradiol and progesterone-induced TFs. **(C)** Yeast two-hybrid AD plasmid expressing GAL4-AD fusion protein by activation of estradiol-induced TF. **(D)** Yeast two-hybrid BD plasmid expressing GAL4-BD fusion protein with FLAG-tag by activation of progesterone-induced TF. L1-9, L1, L14 and L14 represent DNA fragments used for the assembly of the plasmids and correspond with the information in Table S2, Table S3 and Figure S5. MSC represents multi cloning sites. Names of plasmids correspond to those submitted to the Addgene repository.

Table S1. Plasmids used and made in this work.

Vector number	Vector name	Backbone base	Restriction enzymes used to linearize backbone
P1	pHO-UAS::mScarlet-I	HO-Poly-KanMX4-HO (Addgene, MA, USA)	BstEII BamHI
P2	pSite20	HO-Poly-KanMX4-HO (Addgene, MA, USA)	NotI
P3	pSite20 - inducting_TFs	pSite-20 (P2)	KpnI NcoI
P4	pACT-Lex	pACT (Clontech, CA, USA)	ScaI HindIII
P5	pBD-pZ	pBD-GAL4 (Clontech, CA, USA)	NsiI HpaI
P6	pBD-pZ-FLAG	pBD-pZ (P5)	EcoRI SalI

Table S2. Linear fragments used in this work.

Number	Name	Function	length	Source of template for amplification	Primer F	Primer R
L1	UAS	Upstream Activation Sequence (UAS), promoter sequence recognized by GAL4 DNA binding domain	327	Y190 genomic DNA	GTACGCTGCAGGTCGACGCTTCCAAAGCAGGTTGAGAC	CTTTGCCTTCGTTTATCTTGCCT
L2	mScarlet-I	Fluorescent protein	716	Synthetic sequence (Twist Biosciense, CA, USA)	CAAGATAAACGAAGGCAAAGATGGTGAGCAAGGGCGAGGC	CTTGTACAGCTCGTCCATGCC
L3	ADH1 terminator for mScarlet-I	Terminator	248	pBridge (Clontech, CA, USA)	GGCATGGACGAGCTGTACAAGTAGCCAAGCTAATTCCGGGCGAA	GGCATGGACGAGCTGTACAAGTAGCCAAGCTAATTCCGGGCGAA
L4	20 Up	Overlap sequence for genomic integration via homologic recombination	734	Y190 genomic DNA	CGCCACTAGTCCGAGGCCGCCAGGCGCCTTTATATCAT	CCAATAGGATCTCTGGCATGCCATGGTTTGCGAAACCCTATGCTCT
L5	20 Down	Overlap sequence for genomic integration via homologic recombination	692	Y190 genomic DNA	CATGCCAGAGATCCTATTGGGGTACCAATGGAAGGTCGGGATGAG	ATCGATCCACTAGTTCTAGAGCAGCCGCTACCAAACAGACAAGA
L6	ADH1 promoter	Promoter	437	pACT (Clontech, CA, USA)	AACAGAGCATAGGGTTTCGCAAACGCAACTTCTTTTCTTTTTTTTTC	CTTTGCAAAGCTTGGAGTTGAT
L7	LexA-ER-haB112	Estradiol responsive transcription factor that will activate gene expression under regulation of Lex operator	2038	FRP880_PACT1(-1-520)-LexA-ER-haB112-TCYC1 (Addgene)	ATCAACTCCAAGCTTTGCAAAGATGAAAGCGTTAACGGCCAGGC	GGAAAAGGGGCCTGTCTCG
L8	ADH1 terminator-NAT cassette	Terminator + nourseothricin resistance cassette	1804	pAG25 (Addgene, MA, USA)	ATCCTCGAGACAGGCCCCTTTTCCAACTAGTAGCTTTGGACTTCTTC	CCGCCTTTGAGTGAGCTGA

L9	ZPM cassette	Progesterone responsive transcription factor that will activate gene expression under regulation of pZ promoter	3405	pHES830 (Addgene, MA, USA)	GGTATCAGCTCACTCAAAGGCGGCCGCCATCCAGTGTCGAAA	CTCATCCCGACCTTCCATTGGTACGGGAGCCCTTGCATGACAA
L10	backbone-frag1	Fragment needed to restore the plasmid construction after restriction	1809	pACT (Clontech, CA, USA)	TCGCCGCATACACTATTCTCA	GCGTTTCGGTGATGACGG
L11	LexOp x4	Promoter (activated by the estradiol responsive transcription factor)	464	FRP1642_PTEF-HygMX-TTEF-insul-(lexA-box)4-PminCYC1 (Addgene, MA, USA)	TTCACCGTCATCACCGAAACGCTTCGATACTAACGCCGCCA	AATTAATTCCGCTTTATCCATCCTGCAGCCCGGGGGATCCA
L12	backbone-frag2	Fragment needed to restore the plasmid construction after restriction	771	pACT (Clontech, CA, USA)	ATGGATAAAGCGGAATTAATT	ACCTCTGGCGAAGAAGTCC
L13	backbone-frag3	Fragment needed to restore the plasmid construction after restriction	308	pBD-GAL4 (Clontech, CA, USA)	AATAGCGCTCTCGGGATGCA	GCAACGAAGCATCTGTG
L14	pZ promoter	Promoter (activated by the progesteron responsive transcription factor)	742	pHES822 (Addgene, MA, USA)	CACAGATGCTTCGTTGCGCGGTACCGGGCCCTTAT	TTTATTCTCGAGTATAGTTTTTTCTCCT
L15	backbone-frag4	Fragment needed to restore the plasmid construction after restriction	349	pBD-GAL4 (Clontech, CA, USA)	GAAAAAACTATACTCGAGAATAAAATGAAGCTACTGTCTTCTATCGA	CTGTGACGGCATCTTTATTCACA
L16	Flag Oligo	Common epitope	62	Synthetic sequence (IDT, IA, USA)	AATTTGACTACAAGGATGATGACGATAAAGCGGAATTCGCCCGGGCCTCGAGCCCGGG	TCGACCCGGGCTCGAGGCCCGGGCGAATTCCGCTTTATCGTCATCATCCTTGTAGTCA

Table S3. Sequences of linear fragments used in this work.

Number	Name	sequence
L1	UAS	GTACGCTGCAGGTCGACGCTTCCAAAGCAGGTTGAGACAAATCGGATCCGGGGAATTCCCTAGTATTGTAGAATCTTTATTGTTCGGAGCAGTGCGGCGCGAGGCACATCTGCGTTTCAGGAACGCGACCGGTGAAGACGAGGACGCACGGAGGAGAGTCTTCCTTCGGAGGGCTGTCACCCGCTCGGCGGCTTCTAATCCGTGGAATTCCTTTTTTTTTCTTAGCGATTGGCATTATCACATAATGAATTATACATTATATAAAGTAATGTGATTTCTTCGAAGAATATACTAAAAAATGAGCAGGCAAGATAAACGAAGGCAAAG
L2	mScarlet-I	CAAGATAAACGAAGGCAAAGATGGTGAGCAAGGGCGAGGCAGTGATCAAGGAGTTCATGCGGTTCAAGGTGCACATGGAGGGCTCCATGAACGGCCACGAGTTCGAGATCGAGGGCGAGGGCGAGGGCCGCCCCTACGAGGGCACCCAGACCGCCAAGCTGAAGGTGACCAAGGGTGGCCCCCTGCCCTTCTCCTGGGACATCCTGTCCCCTCAGTTCATGTACGGCTCCAGGGCCTTCATCAAGCACCCCGCCGACATCCCCGACTACTATAAGCAGTCCTTCCCCGAGGGCTTCAAGTGGGAGCGCGTGATGAACTTCGAGGACGGCGGCGCCGTGACCGTGACCCAGGACACCTCCCTGGAGGACGGCACCCTGATCTACAAGGTGAAGCTCCGCGGCACCAACTTCCCTCCTGACGGCCCCGTAATGCAGAAGAAGACAATGGGCTGGGAAGCGTCCACCGAGCGGTTGTACCCCGAGGACGGCGTGCTGAAGGGCGACATTAAGATGGCCCTGCGCCTGAAGGACGGCGGCCGTTACCTGGCGGACTTCAAGACCACCTACAAGGCCAAGAAGCCCGTGCAGATGCCCGGCGCCTACAACGTGGACCGCAAGTTGGACATCACCTCCCACAACGAGGACTACACCGTGGTGGAACAGTACGAACGCTCCGAGGGCCGCCACTCCACCGGCGGCATGGACGAGCTGTACAAG
L3	ADH1 terminator for mScarlet-I	GGCATGGACGAGCTGTACAAGTAGCCAAGCTAATTCCGGGCGAATTTCTTATGATTTATGATTTTTATTATTAAATAAGTTATAAAAAAAATAAGTGTATACAAATTTTAAAGTGACTCTTAGGTTTTAAAACGAAAATTCTTGTTCTTGAGTAACTCTTTCCTGTAGGTCAGGTTGCTTTCTCAGGTATAGCATGAGGTCGCTCTTATTGACCACACCTCTACCGGCATCCGGCCAGCGACATGGAG
L4	20 Up	CGCCACTAGTCCGAGGCCGCCAGGCGCCTTTATATCATATAATTAAGACACAAAAGGATAAAACAAAGGTGTTAACTATTCTGCATACTCACTATCGTAAACTGTCCTGCAAATCGTGTAAATATGTATTTCATTTTTTTTGCAGTGAAAAAAGGCATGTAAAATACCGCATCAAGTAACTCTACTCCGCCTGTGGTTTCAAGACTAACGGCTTGAGACAAAATGGGAAGAAATGATTGCAGAAAAGCCATATGTGTAATAGCAAAAAGCTGGATAGTGCTTACCAGATGTTTACCTTAATTTCTTGGTGAATTAGAGAAGTACAGAAGTTTTACTATTAATCCCACCATAGAAATTTGTATAGGAAAGTAGTTTATTGGAGTTATTGGATATACTGTGTAAACTATTTCTTGAAATTGTAATCTTAAGATGCTCTTCTTATTCTATTAAAAATAGAAAATGATTTTCATATTTATTTATTTATTTATATTTTGGCATTACTCTTCATCATTTTTTTCCCTCTAAGAAGCTTCCTTTCTTTTTATAAGGATAACAAAACCAAAAGGAATATTGGGTCAGATGAATGGACGCGAATGCAAGACAGAAGTCCAAATCACGTCAAGACAAAGAAAGAAAGAAAGAAAAACTAACACATTAATGTAGTTTTAAAATTTCAAATCCGAACAACAGAGCATAGGGTTTCGCAAACCATGGCATGCCAGAGATCCTATTGG
L5	20 Down	CATGCCAGAGATCCTATTGGGGTACCAATGGAAGGTCGGGATGAGCATATACAAGCACTAAGAAGAACAATACAGAACTCTACACGGTATTATTGTGCTACAAGCTCGAGTAAAACCGAGTGTTTTGACGATACTAACGTTGTTAAGAAAGTAACTTGTTATCAAACTCATTACCAACTTGTGATTAATTGGTGAATAATATGATAATTGTCGAAATTCCATTGTTGGTAAAGCCTATAATATTATGTATACAGATTATACTAGAAATTCTCTCGAGAATATAAGAATCCCCAAAATTGAATCGGTATTTCTACATACTAATATTACCATTACTTCTCCTTTCGTTTTATATGTTTCATTCCTATTACATTATCGATCTTTGCATTTCAGCTTCCATTATATTTGATGTCTGTTTTATGTCCCCACGTTACACCGCATGTGACAGTATACTAGTAACATGAGTGCTACCGAATAGATGACATTTTAGACTTTCATTCCAACAACTTGGTTGACAGAATGTTACGTACCCTATATCTAATCTATATGAGGCCTGAATCTAACTGAAAGGTGGAATTTCAGTAATTTATCAAGCTTTAATAAGTTTGGGTAGTTTAACTGTGCAAAAAGGTATTTACCTTACATACTGAATCTTGTCTGTTTGGTAGCGGCTGCTCTAGAACTAGTGGATCGAT
L6	ADH1 promoter	AACAGAGCATAGGGTTTCGCAAACGCAACTTCTTTTCTTTTTTTTTCTTTTCTCTCTCCCCCGTTGTTGTCTCACCATATCCGCAATGACAAAAAAAAATGATGGAAGACACTAAAGGAAAAAATTAACGACAAAGACAGCACCAACAGATGTCGTTGTTCCAGAGCTGATGAGGGGTATCTTCGAACACACGAAACTTTTTCCTTCCTTCATTCACGCACACTACTCTCTAATGAGCAACGGTATACGGCCTTCCTTCCAGTTACTTGAATTTGAAATAAAAAAAGTTTGCCGCTTTGCTATCAAGTATAAATAGACCTGCAATTATTAATCTTTTGTTTCCTCGTCATTGTTCTCGTTCCCTTTCTTCCTTGTTTCTTTTTCTGCACAATATTTCAAGCTATACCAAGCATACAATCAACTCCAAGCTTTGCAAAG
L7	LexA-ER-haB112	ATCAACTCCAAGCTTTGCAAAGATGAAAGCGTTAACGGCCAGGCAACAAGAGGTGTTTGATCTCATCCGTGATCACATCAGCCAGACAGGTATGCCGCCGACGCGTGCGGAAATCGCGCAGCGTTTGGGGTTCCGTTCCCCAAACGCGGCTGAAGAACATCTGAAGGCGCTGGCACGCAAAGGCGTTATTGAAATTGTTTCCGGCGCATCACGCGGGATTCGTCTGTTGCAGGAAGAGGAAGAAGGGTTGCCGCTGGTAGGTCGTGTGGCTGCCGGTGAACCACTTCTGGCGCAACAGCATATTGAAGGTCATTATCAGGTCGATCCTTCCTTATTCAAGCCGAATGCTGATTTCCTGCTGCGCGTCAGCGGGATGTCGATGAAAGACATCGGCATTATGGATGGTGACTTGCTGGCAGTGCATAAAACTCAGGATGTACGTAACGGT

		CAGGTCGTTGTCGCACGTATTGATGACGAGGTTACCGTTAAGCGCCTGAAAAAACAGGGCAATAAAGTCGAACTGTTGCCAGAAAATAGCGAGTTTAAACCAATTGTCGTAG<br>ATCTTCGTCAGCAGAGCTTCACCATTGAAGGGCTGGCGGTTGGGGTTATTCGCAACGGCGACTGGCTGGGTTCTAAGGATATCTCTGCTGGAGACATGAGAGCTGCCAACCT<br>TTGGCCAAGCCCGCTCATGATCAAACGCTCTAAGAAGAACAGCCTGGCCTTGTCCCTGACGGCCGACCAGATGGTCAGTGCCTTGTTGGATGCTGAGCCCCCCATACTCTATTC<br>CGAGTATGATCCTACCAGACCCTTCAGTGAAGCTTCGATGATGGGCTTACTGACCAACCTGGCAGACAGGGAGCTGGTTCACATGATCAACTGGGCGAAGAGGGTGCCAGGC<br>TTTGTGGATTTGACCCTCCATGATCAGGTCCACCTTCTAGAATGTGCCTGGTTAGAGATCCTGATGATTGGTCTCGTCTGGCGCTCCATGGAGCACCCAGTGAAGCTACTGTTT<br>GCTCCTAACTTGCTCTTGGACAGGAACCAGGGAAAATGTGTAGAGGGCATGGTGGAGATCTTCGACATGCTGCTGGCTACATCATCTCGGTTCCGCATGATGAATCTGCAGG<br>GAGAGGAGTTTGTGTGCCTCAAATCTATTATTTTGCTTAATTCTGGAGTGTACACATTTCTGTCCAGCACCCTGAAGTCTCTGGAAGAGAAGGACCATATCCACCGAGTCCTGG<br>ACAAGATCACAGACACTTTGATCCACCTGATGGCCAAGGCAGGCCTGACCCTGCAGCAGCAGCACCAGCGGCTGGCCCAGCTCCTCCTCATCCTCTCCCACATCAGGCACATG<br>AGTAACAAAGGCATGGAGCATCTGTACAGCATGAAGTGCAAGAACGTGGTGCCCCTCTATGACCTGCTGCTGGAGATGCTGGACGCCCACCGCCTACATGCGCCCACTAGCC<br>GTGGAGGGGCATCCGTGGAGGAGACGGACCAAAGCCACTTGGCCACTGCGGGCTCTACTTCATCGCATTCCTTGCAAAAGTATTACATCACGGGGGAGGCAGAGGGTTTCCC<br>TGCCACAGTCGATATCATGCAGGATCTGCCGGGCAACGATAACAGCACCGCGGGCGAATTTCCAGGTATTACTTTGAGAATCCAAGAAACTGATATGTTGTATAAAGGTGATA<br>CTTTGTATTTGGATTGGTTGGAAGATGGTATTGCTGAATTGGTTTTCGATGCTCCAGGTTCTGTTAATAAATTGGATACTGCTGTTGCTTCTTTGGGTGAAGCTATTGGTGTTTT<br>GGAACAACAATCTGATTTGATTTGGGAAACTTTGACTGTTAAAGATGCTAAAGTTAATTTCGATTCTGGTTTGGAAAAATTCGAAGAAGCTATTCCATCTGCTGATGATTTCGA<br>TCCAGTTGCTGAAAGAAGATCCTCTGGTGAATTTAGAGCTGAAAGACATTCTGGTGGTACTGATTTGTGTTTCAAGCTTTAAACGCGTTTAAAGCTTATCCTCGAGACAGGCCC<br>CTTTTCC
L8	ADH1 terminator-NAT cassette	ATCCTCGAGACAGGCCCCTTTTCCAACTAGTAGCTTTGGACTTCTTCGCCAGAGGTTTGGTCAAGTCTCCAATCAAGGTTGTCGGCTTGTCTACCTTGCCAGAAATTTACGAAAA<br>GATGGAAAAGGGTCAAATCGTTGGTAGATACGTTGTTGACACTTCTAAATAAGCGAATTTCTTATGATTTATGATTTTTATTATTAAATAAGTTATAAAAAAAATAAGTGTATAC<br>AAATTTTAAAGTGACTCTTAGGTTTTAAAACGAAAATTCTTATTCTTGAGTAACTCTTTCCTGTAGGTCAGGTTGCTTTCTCAGGTATAGCATGAGGTCGCTCTTATTGACCACAC<br>CTCTACCGGCATGAGATCTGTTTAGCTTGCCTTGTCCCCGCCGGGTCACCCGGCCAGCGACATGGAGGCCCAGAATACCCTCCTTGACAGTCTTGACGTGCGCAGCTCAGGGG<br>CATGATGTGACTGTCGCCCGTACATTTAGCCCATACATCCCCATGTATAATCATTTGCATCCATACATTTTGATGGCCGCACGGCGCGAAGCAAAAATTACGGCTCCTCGCTGC<br>AGACCTGCGAGCAGGGAAACGCTCCCCTCACAGACGCGTTGAATTGTCCCCACGCCGCGCCCCTGTAGAGAAATATAAAAGGTTAGGATTTGCCACTGAGGTTCTTCTTTCAT<br>ATACTTCCTTTTAAAATCTTGCTAGGATACAGTTCTCACATCACATCCGAACATAAACAACCATGGGTACCACTCTTGACGACACGGCTTACCGGTACCGCACCAGTGTCCCGG<br>GGGACGCCGAGGCCATCGAGGCACTGGATGGGTCCTTCACCACCGACACCGTCTTCCGCGTCACCGCCACCGGGGACGGCTTCACCCTGCGGGAGGTGCCGGTGGACCCGC<br>CCCTGACCAAGGTGTTCCCCGACGACGAATCGGACGACGAATCGGACGCCGGGGAGGACGGCGACCCGGACTCCCGGACGTTCGTCGCGTACGGGGACGACGGCGACCTG<br>GCGGGCTTCGTGGTCGTCTCGTACTCCGGCTGGAACCGCCGGCTGACCGTCGAGGACATCGAGGTCGCCCCGGAGCACCGGGGGCACGGGGTCGGGCGCGCGTTGATGGG<br>GCTCGCGACGGAGTTCGCCCGCGAGCGGGGCGCCGGGCACCTCTGGCTGGAGGTCACCAACGTCAACGCACCGGCGATCCACGCGTACCGGCGGATGGGGTTCACCCTCTG<br>CGGCCTGGACACCGCCCTGTACGACGGCACCGCCTCGGACGGCGAGCAGGCGCTCTACATGAGCATGCCCTGCCCCTAATCAGTACTGACAATAAAAAGATTCTTGTTTTCAA<br>GAACTTGTCATTTGTATAGTTTTTTTATATTGTAGTTGTTCTATTTTAATCAAATGTTAGCGTGATTTATATTTTTTTTCGCCTCGACATCATCTGCCCAGATGCGAAGTTAAGTGC<br>GCAGAAAGTAATATCATGCGTCAATCGTATGTGAATGCTGGTCGCTATACTGCTGTCGATTCGATACTAACGCCGCCATCCAGTGTCGAAAACGAGCTCGAATTCATCGATGA<br>TATCAGATCCACTAGTGGCCTATGCGGCCGCGGATCTGCCGGTCTCCCTATAGTGAGTCGTATTAATTTCGATAAGCCAGGTTAACCTGCATTAATGAATCGGCCAACGCGCG<br>GGGAGAGGCGGTTTGCGTATTGGGCGCTCTTCCGCTTCCTCGCTCACTGACTCGCTGCGCTCGGTCGTTCGGCTGCGGCGAGCGGTATCAGCTCACTCAAAGGCGG
L9	ZPM cassette	GGTATCAGCTCACTCAAAGGCGGCCGCCATCCAGTGTCGAAAACGACCTCGAATTCATCGAGTGGATTCGGCTTTGGGTACCGGGCCCGGCAACCAAACCCATACATCGGGA<br>TTCCTATAATACCTTCGTTGGTCTCCCTAACATGTAGGTGGCGGAGGGGAGATATACAATAGAACAGATACCAGACAAGACATAATGGGCTAAACAAGACTACACCAATTACA<br>CTGCCTCATTGATGGTGGTACATAACGAACTAATACTGTAGCCCTAGACTTGATAGCCATCATCATATCGAAGTTTCACTACCCTTTTTCCATTTGCCATCTATTGAAGTAATAAT<br>AGGCGCATGCAACTTCTTTTCTTTTTTTTTCTTTTCTCTCTCCCCCGTTGTTGTCTCACCATATCCGCAATGACAAAAAAATGATGGAAGACACTAAAGGAAAAAATTTAACGACAA<br>AGACAGCACCAACAGATGTCGTTGTTCCAGAGCTGATGAGGGGTATCTCGAAGCACACGAAACTTTTTCCTTCCTTCATTCACGCACACTACTCTCTAATGAGCAACGGTATAC<br>GGCCTTCCTTCCAGTTACTTGAATTTGAAATAAAAAAAAGTTTGCTGTCTTGCTATCAAGTATAAATAGACCTGAATCCCTATCAGTGATAGAGATCTCCCTATCAGTGATAGAG<br>ATTCTTCCTTGTTTCTTTTTCTGCAGGCTAGCAATAAAATGGGTACCCGCCCATATGCTTGCCCTGTCGAGTCCTGCGATCGCCGCTTTTCTCGCTCGGATGAGCTTACCCGCCAT<br>ATCCGCATCCATACCGGTCAGAAGCCCTTCCAGTGTCGAATCTGCATGCGTAACTTCAGTCGTAGTGACCACCTTACCACCCACATCCGCACCCACACAGGCGAGAAGCCTTTT

		GCCTGTGACATTTGTGGGAGGAAGTTTGCCAGGAGTGATGAACGCAAGAGGCATACCAAAATCCATACAGGTAGATCCGGTGACGGTGCTGGTTTAAAGTTCAATAAAGTCA<br>GAGTTGTGAGAGCACTGGATGCTGTTGCTCTCCCACAGCCATTGGGCGTTCCAAATGAAAGCCAAGCCCTAAGCCAGAGATTCACTTTTTCACCAGGTCAAGACATACAGTTG<br>ATTCCACCACTGATCAACCTGTTAATGAGCATTGAACCAGATGTGATCTATGCAGGACATGACAACACAAAACCTGACACCTCCAGTTCTTTGCTGACAAGTCTTAATCAACTA<br>GGCGAGAGGCAACTTCTTTCAGTAGTCAAGTGGTCTAAATCATTGCCAGGTTTTCGAAACTTACATATTGATGACCAGATAACTCTCATTCAGTATTCTTGGATGAGCTTAATG<br>GTGTTTGGTCTAGGATGGAGATCCTACAAACATGTCAGTGGGCAGATGCTGTATTTTGCACCTGATCTAATACTAAATGAACAGCGGATGAAAGAATCATCATTCTATTCATTA<br>TGCCTTACCATGTGGCAGATCCCACAGGAGTTTGTCAAGCTTCAAGTTAGCCAAGAAGAGTTCCTCTGTATGAAAGTATTGTTACTTCTTAATACAATTCCTTTGGAAGGGCTA<br>CGAAGTCAAACCCAGTTTGAGGAGATGAGGTCAAGCTACATTAGAGAGCTCATCAAGGCAATTGGTTTGAGGCAAAAAGGAGTTGTGTCGAGCTCACAGCGTTTCTATCAAC<br>TTACAAAACTTCTTGATAACTCGCATGATCTTGTCAAACAGCTTCATCTGTACTGCTTGAATACATTTATCCAGTCCCGGGCACTGAGTGTTGAATTTCCAGAAATGATGTCTGA<br>AGTTATTGCTGCACAATTACCCAAGATATTGGCAGGGATGGTGAAACCCCTTCTCTTTCATAAAAAGGCGGCCGCAGGTGACGGTGCTGGTTTAATTAACATGACGGTCGACC<br>ATGATTTCAATAGCGAAGATATTTTATTCCCCATAGAAAGCATGAGTAGTATACAATACGTGGAGAATAATAACCCAAATAATATTAACAACGATGTTATCCCGTATTCTCTAG<br>ATATCAAAAACACTGTCTTAGATAGTGCGGATCTCAATGACATTCAAAATCAAGAAACTTCACTGAATTTGGGGCTTCCTCCACTATCTTTCGACTCTCCACTGCCCGTAACGGA<br>AACGATACCATCCACTACCGATAACAGCTTGCATTTGAAAGCTGATAGCAACAAAAATCGCGATGCAAGAACTATTGAAAATGATAGTGAAATTAAGAGTACTAATAATGCTA<br>GTGGCTCTGGGGCAAATCAATACACAACTCTTACTTCACCTTATCCTATGAACGACATTTTGTACAACATGAACAATCCGTTACAATCACCGTCACCTTCATCGGTACCTCAAAA<br>TCCGACTATAAATCCTCCCATAAATACAGCAAGTAACGAAACTAATTTATCGCCTCAAACTTCAAATGGTAATGAAACTCTTATATCTCCTCGAGCCCAACAACATACGTCCATT<br>AAAGATAATCGTCTGTCCTTACCTAATGGTGCTAATTCGAATCTTTTCATTGACACTAACCCAAACAATTTGAACGAAAAACTAAGAAATCAATTGAACTCAGATACAAATTCAT<br>ATTCTAACTCCATTTCTAATTCAAACTCCAATTCTACGGGTAATTTAAATTCCAGTTATTTTAATTCACTGAACATAGACTCCATGCTAGATGATTACGTTTCTAGTGATCTCTTAT<br>TGAATGATGATGATGATGACACTAATTTATCACGCCGAAGATTTAGCGACGTTATAACAAACCAAGAGCTCTAAGCAAATAGCTAAATTATATACGAATTAATATTATGATTAA<br>GTGTTTACGTGAGTGCGATATTTTTATTACTATCTTATACAGTTGTATATACTCTATAAAATGAGTTGTCTATTAATTAACGCGATGAATGCTTTCTGGGTTTACCTCTCCAACAA<br>CTCTAGTTTACTTCTCAATACATTCAATTGTATTTGATTTGTCAATACTTCATCATTAATCAATTCTATAGTTTTGTTTTTCTCGTTTATTTCCAAATTTAATGCATCAATTTTATTAT<br>TCAATTTGTCGTTGATTTTGGTTAATGATTTTATGGTTTGATCTCTGGCATTGATTGTTTGTGTTAGTTTTTCATTATTGATAATTAAATTATTTAAGTTAGTTATCAACTCGGTGT<br>TTTCAAGTTTCAAGTTTTCAATTTCTTTAGAGTTTATTAGATTTGTCAAAGTTTCTGAATTGCTTGATTGGTCCGGCCCAGGTATTGTTAGCGGTTTGAAGCAGGCGGCAGAAGA<br>AGTAACAAAGGAACCTAGAGGCCTTTTGATGTTAGCAGAATTGTCATGCAAGGGCTCCCGTACCAATGGAAGGTCGGGATGAG
L10	backbone-frag1	TCGCCGCATACACTATTCTCAGAATGACTTGGTTGAGTACTCACCAGTCACAGAAAAGCATCTTACGGATGGCATGACAGTAAGAGAATTATGCAGTGCTGCCATAACCATGA<br>GTGATAACACTGCGGCCAACTTACTTCTGACAACGATCGGAGGACCGAAGGAGCTAACCGCTTTTTTGCACAACATGGGGGATCATGTAACTCGCCTTGATCGTTGGGAACCG<br>GAGCTGAATGAAGCCATACCAAACGACGAGCGTGACACCACGATGCCTGCAGCAATGGCAACAACGTTGCGCAAACTATTAACTGGCGAACTACTTACTCTAGCTTCCCGGCA<br>ACAATTAATAGACTGGATGGAGGCGGATAAAGTTGCAGGACCACTTCTGCGCTCGGCCCTTCCGGCTGGCTGGTTTATTGCTGATAAATCTGGAGCCGGTGAGCGTGGGTCT<br>CGCGGTATCATTGCAGCACTGGGGCCAGATGGTAAGCCCTCCCGTATCGTAGTTATCTACACGACGGGGAGTCAGGCAACTATGGATGAACGAAATAGACAGATCGCTGAGA<br>TAGGTGCCTCACTGATTAAGCATTGGTAACTGTCAGACCAAGTTTACTCATATATACTTTAGATTGATTTAAAACTTCATTTTTAATTTAAAAGGATCTAGGTGAAGATCCTTTTT<br>GATAATCTCATGACCAAAATCCCTTAACGTGAGTTTTCGTTCCACTGAGCGTCAGACCCCGTAGAAAAGATCAAAGGATCTTCTTGAGATCCTTTTTTTCTGCGCGTAATCTGCT<br>GCTTGCAAACAAAAAAACCACCGCTACCAGCGGTGGTTTGTTTGCCGGATCAAGAGCTACCAACTCTTTTTCCGAAGGTAACTGGCTTCAGCAGAGCGCAGATACCAAATACT<br>GTCCTTCTAGTGTAGCCGTAGTTAGGCCACCACTTCAAGAACTCTGTAGCACCGCCTACATACCTCGCTCTGCTAATCCTGTTACCAGTGGCTGCTGCCAGTGGCGATAAGTCG<br>TGTCTTACCGGGTTGGACTCAAGACGATAGTTACCGGATAAGGCGCAGCGGTCGGGCTGAACGGGGGGTTCGTGCACACAGCCCAGCTTGGAGCGAACGACCTACACCGAA<br>CTGAGATACCTACAGCGTGAGCTATGAGAAAGCGCCACGCTTCCCGAAGGGAGAAAGGCGGACAGGTATCCGGTAAGCGGCAGGGTCGGAACAGGAGAGCGCACGAGGG<br>AGCTTCCAGGGGGAAACGCCTGGTATCTTTATAGTCCTGTCGGGTTTCGCCACCTCTGACTTGAGCGTCGATTTTTGTGATGCTCGTCAGGGGGGCGGAGCCTATGGAAAAAC<br>GCCAGCAACGCGGCCTTTTTACGGTTCCTGGCCTTTTGCTGGCCTTTTGCTCACATGTTCTTTCCTGCGTTATCCCCTGATTCTGTGGATAACCGTATTACCGCCTTTGAGTGAGC<br>TGATACCGCTCGCCGCAGCCGAACGACCGAGCGCAGCGAGTCAGTGAGCGAGGAAGCGGAAGAGCGCCTGATGCGGTATTTTCTCCTTACGCATCTGTGCGGTATTTCACAC<br>CGCATATGGTGCACTCTCAGTACAATCTGCTCTGATGCCGCATAGTTAAGCCAGTATACACTCCGCTATCGCTACGTGACTGGGTCATGGCTGCGCCCCGACACCCGCCAACAC<br>CCGCTGACGCGCCCTGACGGGCTTGTCTGCTCCCGGCATCCGCTTACAGACAAGCTGTGACCGTCTCCGGGAGCTGCATGTGTCAGAGGTTTTCACCGTCATCACCGAAACGC

L11	LexOp x4	TTCACCGTCATCACCGAAACGCTTCGATACTAACGCCGCCATCCAGTGTTTAAACGAACTAGTGCGGCCGCAATAATATATAAACCTGTATAATATAACCTTGAAGACTATATTTCTTTTCGAGCTCCCTAGGTGCTGTATATACTCACAGCATAACTGTATATACACCCAGGGTCTAGGTGCTGTATATACTCACAGCATAACTGTATATACACCCAGGGTCTAGGTGCTGTATATACTCACAGCATAACTGTATATACACCCAGGGTCTAGGTGCTGTATATACTCACAGCATAACTGTATATACACCCAGGGTCTAGAGCATGTGCTCTGTATGTATATAAAACTCTTGTTTTCTTCTTTTCTCTTCTTTCCTTATACATTAGGACCTTTGCAGCATAAATTACTATACTTCTATACTAGTGGATCCCCCGGGCTGCAGGATGGATAAAGCGGAATTAATT
L12	backbone-frag2	ATGGATAAAGCGGAATTAATTCCCGAGCCTCCAAAAAAGAAGAGAAAGGTCGAATTGGGTACCGCCGCCAATTTTAATCAAAGTGGGAATATTGCTGATAGCTCATTGTCCTTCACTTTCACTAACAGTAGCAACGGTCCGAACCTCATAACAACTCAAACAAATTCTCAAGCGCTTTCACAACCAATTGCCTCCTCTAACGTTCATGATAACTTCATGAATAATGAAATCACGGCTAGTAAAATTGATGATGGTAATAATTCAAAACCACTGTCACCTGGTTGGACGGACCAAACTGCGTATAACGCGTTTGGAATCACTACAGGGATGTTTAATACCACTACAATGGATGATGTATATAACTATCTATTCGATGATGAAGATACCCCACCAAACCCAAAAAAAGAGATCTGTATGGCTTACCCATACGATGTTCCAGATTACGCTAGCTTGGGTGGTCATATGGCCATGGAGGCCCCGGGGATCCGAATTCGAGCTCGAGAGATCTATGAATCGTAGATACTGAAAAACCCCGCAAGTTCACTTCAACTGTGCATCGTGCACCATCTCAATTTCTTTCATTTATACATCGTTTTGCCTTCTTTTATGTAACTATACTCCTCTAAGTTTCAATCTTGGCCATGTAACCTCTGATCTATAGAATTTTTTAAATGACTAGAATTAATGCCCATCTTTTTTTTGGACCTAAATTCTTCATGAAAATATATTACGAGGGCTTATTCAGAAGCTTTGGACTTCTTCGCCAGAGGT
L13	backbone-frag3	AATAGCGCTCTCGGGATGCATTTTTGTAGAACAAAAAAGAAGTATAGATTCTTTGTTGGTAAAATAGCGCTCTCGCGTTGCATTTCTGTTCTGTAAAAATGCAGCTCAGATTCTTTGTTTGAAAAATTAGCGCTCTCGCGTTGCATTTTTGTTTTACAAAAATGAAGCACAGATTCTTCGTTGGTAAAATAGCGCTTTCGCGTTGCATTTCTGTTCTGTAAAAATGCAGCTCAGATTCTTTGTTTGAAAAATTAGCGCTCTCGCGTTGCATTTTTGTTCTACAAAATGAAGCACAGATGCTTCGTTGC
L14	pZ promoter	CACAGATGCTTCGTTGCGCGGTACCGGGCCCTTATATTGAATTTTCAAAAATTCTTACTTTTTTTTTGGATGGACGCAAAGAAGTTTAATAATCATATTACATGGCATTACCACCATATACATATCCATATACATATCCATATCTAATCTTACTTATATGTTGTGGAAATGTAAAGAGCCCCATTATCTTAGCCTAAAAAAACCTTCTCTTTGGAACTTTCAGTAATACGCTTAACTGCTCATTGCTATATTGAAGTGCGGCCGCGTGGGCGTGCGTGGGCGGGCGTGGGCGTGCGTGGGCGGGCGTGGGCGTGCGTGGGCGTCTAGACCGTGCGTCCTCGTCTTCACCGGTCGCGTTCCTGAAACGCAGATGTGCCTAATGCCGCACTGCTCCGAACAATAAAGATTCTACAATACTAGCTTTTATGGTTATGAAGAGGAAAAATTGGCAGTAACCTGGCCCCACAAACCTTCAAATTAACGAATCAAATTAACAACCATAGGATGATAATGCGATTAGTTTTTTAGCCTTATTTCTGGGGTAATTAATCAGCGAAGCGATGATTTTTGATCTATTAACAGATATATAAATGGAAAAGCTGCATAACCACTTTAACTAATACTTTCAACATTTTCAGTTTGTATTACTTCTTATTCAAATGTCATAAAAGTATCAACAAAAAATTGTTAATATACCTCTATACTTTAACGTCAAGGAGAAAAAACTATACTCGAGAATAAA
L15	backbone-frag4	GAAAAAACTATACTCGAGAATAAAATGAAGCTACTGTCTTCTATCGAACAAGCATGCGATATTTGCCGACTTAAAAAGCTCAAGTGCTCCAAAGAAAAACCGAAGTGCGCCAAGTGTCTGAAGAACAACTGGGAGTGTCGCTACTCTCCCAAAACCAAAAGGTCTCCGCTGACTAGGGCACATCTGACAGAAGTGGAATCAAGGCTAGAAAGACTGGAACAGCTATTTCTACTGATTTTTCCTCGAGAAGACCTTGACATGATTTTGAAAATGGATTCTTTACAGGATATAAAAGCATTGTTAACAGGATTATTTGTACAAGATAATGTGAATAAAGATGCCGTCACAG
L16	Flag Oligo	AATTTGACTACAAGGATGATGACGATAAAGCGGAATTCGCCCGGGCCTCGAGCCCGGGTCGA
